# Hydrodynamic flow and benthic boundary layer interactions shape the microbial community in Milos shallow water hydrothermal vents

**DOI:** 10.3389/fmicb.2025.1649514

**Published:** 2025-08-29

**Authors:** Ana Clara Pelliciari Silva, Flavia Migliaccio, Bernardo Barosa, Luigi Gallucci, Mustafa Yücel, Dionysis Foustoukos, Nadine Le Bris, Stuart J. Bartlett, Valerio D’Alessandro, Costantino Vetriani, Donato Giovannelli

**Affiliations:** ^1^Department of Biology, University of Naples “Federico II”, Naples, Italy; ^2^Department of Molecular Ecology, Max Planck Institute for Marine Microbiology, Bremen, Germany; ^3^Institute of Marine Sciences, Middle East Technical University, Mersin, Türkiye; ^4^Earth and Planets Laboratory, Carnegie Institution of Washington, Washington, DC, United States; ^5^Institute of Systematics Biodiversity and Evolution, Sorbonne University, Paris, France; ^6^Division of Geological and Planetary Sciences, California Institute of Technology, Pasadena, CA, United States; ^7^Industrial Engineering and Mathematical Sciences Department, Polytechnic University of Marche, Ancona, Italy; ^8^Department of Marine and Coastal Science, Rutgers University, New Brunswick, NJ, United States; ^9^Department of Biochemistry and Microbiology, Rutgers University, New Brunswick, NJ, United States; ^10^National Research Council-Institute of Marine Biological Resources and Biotechnologies (CNR-IRBIM), Ancona, Italy; ^11^Marine Chemistry and Geochemistry Department, Woods Hole Oceanographic Institution, Woods Hole, MA, United States; ^12^Earth-Life Science Institute, Tokyo Institute of Technology, Tokyo, Japan

**Keywords:** shallow-water hydrothermal vents, microbial diversity, metagenomics, fluid-mixing, microbiome, ripples, storms, benthic boundary layer

## Abstract

In shallow-water hydrothermal vents, the dynamic interface between the discharged reduced hydrothermal fluids and the oxidized seawater allows the establishment of gradients capable of supporting diverse and complex microbial mats. Due to their shallow depths and proximity to land masses, shallow vents are heavily influenced by dynamic forcing, tidal fluctuations, and episodic events (e.g., storms, tides, etc.). Although several studies have investigated the microbial communities inhabiting shallow vents in the last decades, less is known about how microbial communities respond to episodic events and how the complex interplay of physical and chemical drivers shapes the establishment and structure of microbial biofilms in these systems. Here we present data combining the taxonomic and functional diversity of the white microbial mats commonly found in sulfide rich shallow-water hydrothermal vents in Paleochori Bay (Milos Island, Greece), using a combined approach of 16S rRNA transcript amplicon sequencing (from RNA) and shotgun metagenomic sequencing (from which 16S rRNA genes were retrieved). We show that the white microbial mats of Milos shallow-water hydrothermal vents are dominated by Epsilonproteobacteria, now classified as Campylobacterota, with metabolic functions associated with chemolithoautotrophic lifestyles and exposed to a diverse array of viral communities. Taxonomic names follow the classification available at the time of analysis (2012). We explore how dynamic forcing and storm events influence microbial community restructuring and turn-over, and provide evidence that dynamic interactions with the benthic boundary layer play a key role in controlling the spatial distribution of taxa. Overall, our results show diverse processes through which geodynamic events influence microbial taxonomic and functional diversity.

## 1 Introduction

Shallow-water hydrothermal vents (SWHVs) are dynamic environments that form near tectonic plate boundaries and active volcanic areas, where geothermal heat drives the circulation of hydrothermal fluids (Price and Giovannelli, 2017). Upon discharge, hydrothermal fluids show unique chemical compositions, and are often enriched in carbon dioxide, sulfide, hydrogen, and iron among other compounds. Several of the compounds and elements released in the water column by SWHV, like iron, cobalt and reduced sulfur and nitrogen compounds, play a crucial role in marine ecosystems, supporting primary productivity and other metabolisms (Yücel et al., 2011; Price and Giovannelli, 2017; Schine et al., 2021; Gledhill and Buck, 2012; Frawley and Fang, 2014; Gomez-Saez et al., 2017). Additionally, the presence of sunlight in SWHVs fosters a dual-energy ecosystem, supporting both photosynthetic and chemosynthetic metabolisms. The availability of locally produced organic carbon, along with terrigenous and water column derived organic matter inputs, support complex heterotrophic microbial communities (Bellec et al., 2020; Arcadi et al., 2023).

Due to their shallow depths, SWHVs are also subject to unique dynamic physical and chemical forces that differ from their deep-sea counterparts. These include increased exposure to storm-induced turbulence, which enhances seawater–hydrothermal fluid mixing and creates steep chemical and thermal gradients across relatively short spatial scales (Yücel et al., 2013). For example, episodic storm events, driven by high wind velocity and surface shear stress, and resulting strong waves, can resuspend sediments in the Milos shallow vent system, altering fluid discharge patterns, and influencing both microbial and geochemical zonation (Yücel et al., 2013). These mixing processes result in the formation of localized microenvironments characterized by rapid changes in temperature, redox conditions, and electron donor/acceptor availability (Yücel, 2013). Such steep gradients strongly influence microbial colonization and niche differentiation. The thermodynamic disequilibria created at the interface of hydrothermal fluids and oxygenated seawater provide abundant energy sources for microbial metabolism, particularly for chemolithoautotrophs that exploit reduced compounds such as hydrogen and sulfide (Price and Giovannelli, 2017; Lu et al., 2020). As a result, SWHVs serve as natural laboratories for studying microbial processes shaped by both geochemical potential and hydrodynamic forces.

Biofilm formation is expected to be a dominant microbial strategy in shallow-water hydrothermal vents, given the dynamic mixing processes and steep physicochemical gradients described above. Biofilms and microbial mats are structured microbial communities embedded in self-produced extracellular polymeric substances that facilitate surface colonization, metabolic cooperation, and resilience to fluctuating environmental conditions (Dobretsov, 2009; Flemming and Wingender, 2010). While extensively studied in deep-sea hydrothermal vents, where they play critical roles in primary productivity and community stability under extreme thermal and chemical regimes (Alain et al., 2004; Gulmann et al., 2015; O’Brien et al., 2015), biofilms are also recognized as key features of microbial life in terrestrial hot springs, such as those in Yellowstone and Iceland, where similarly steep gradients drive spatial microbial stratification (Schuler et al., 2017). In shallow-water hydrothermal vents, however, these structures are likely even more significant due to increased environmental variability, including exposure to light, turbulence, and storm-induced mixing (Price and Giovannelli, 2017; Patwardhan et al., 2018; Sciutteri et al., 2022). Shifting physical and chemical conditions in SWHV selects for microbial assemblages capable of adhering to surfaces and rapidly adapting to changing redox and nutrient conditions, making biofilms not just prevalent but essential to microbial survival and ecosystem function in shallow vents.

**Figure 1:**
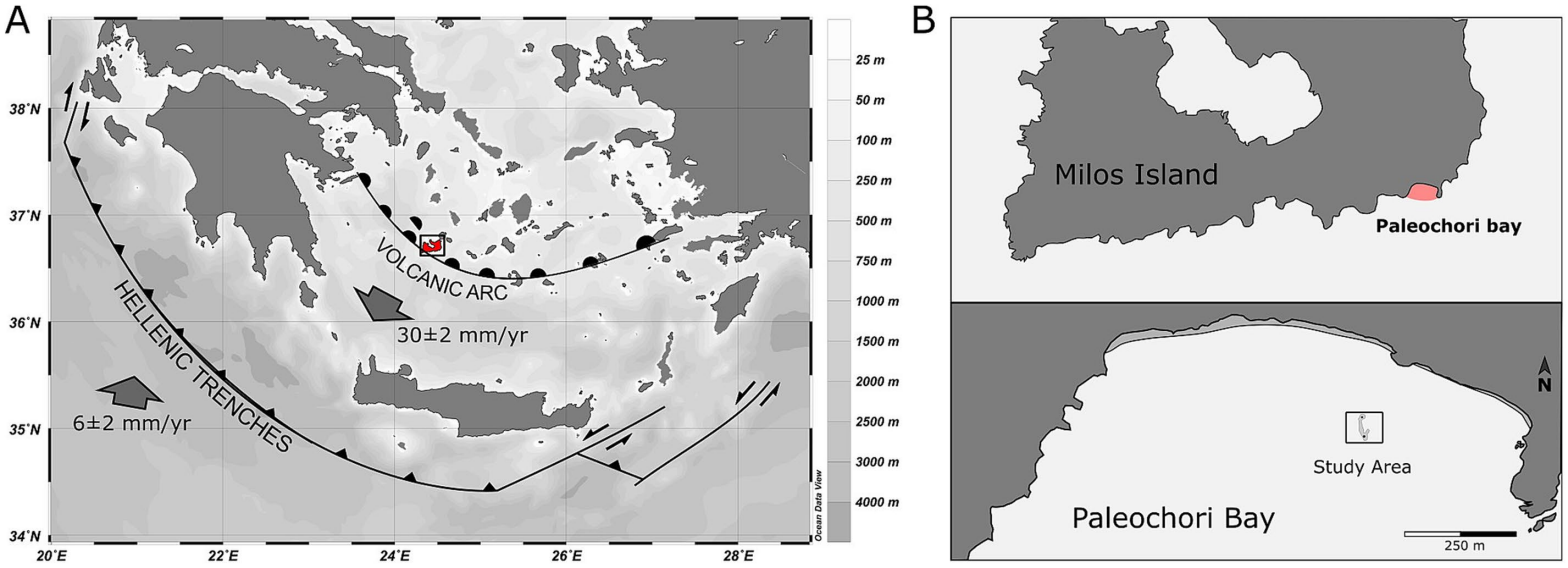
**(A)** Map of the Eastern Mediterranean Sea and Aegean Sea showing the position of the Hellenic Trenches and the Hellenic Volcanic Arc (adapted from Kokkalas et al., 2006). **(B)** Upper: Map of the south portion of Milos Island; lower: detail of the study area in Paleochori Bay.

The hydrothermal area of Milos Island, situated within the Aegean Volcanic Arc, is a region extending from the island of Kos close to the Turkish coast in the east to Methana in the west. The arc has been formed by the subduction of the African plate beneath the northeastern edge of the European plate, hosting Milos Island. Milos island spans a geo-active zone of vents covering approximately 35 km^2^ and stands as one of the most extensively studied shallow-water geothermal systems globally (Dando et al., 1995, 2000; Price and Giovannelli, 2017; Nomikou et al., 2021). Within this area, Paleochori Bay, located on the southeast coast of Milos island (Figure 1), represents a venting site characterized by intense degassing, hydrothermal activity, and a pronounced thermal and redox vertical gradient (Dando et al., 1995, 2000; Yücel et al., 2013).

The entire Paleochori Bay area manifests as regions of elevated temperature and degassing on the sandy seabed (Khimasia et al., 2020). Vertical gradients in temperature and redox are steep, with sediment depth correlating with temperature rise, whereas horizontal changes in temperature and redox are gradual and decrease as the distance from the venting center increases (Sievert et al., 1999; Sievert et al., 2000; Dando et al., 2000; Wenzhöfer et al., 2000). Reported temperatures of up to 119 °C at a vent site at 10-meter water depth underscore the extreme thermal conditions present (Botz et al., 1996). Venting fluids exhibit varying salinity levels, often mixed with freshwater. The CO_2_ content in these fluids ranges between 54.9 and 91.9%, while the emissions of H_2_S, CH_4_ and H_2_ comprise ≤ 8.1%, ≤ 9.7%, and ≤ 3%, respectively (Botz et al., 1996; Dando et al., 2000). Hydrothermal fluids have been found to contain elevated concentrations of reduced inorganic chemicals, including NH_4_^+^ (up to 1 mM), Mn^2+^ (up to 0.4 mM) (Fitzsimons et al., 1997), and arsenic primarily in the trivalent oxidation state [As(III)] (in the range of 30 uM) (Price et al., 2013). Common occurrences near vent orifices include arsenic minerals and elemental sulfur precipitates, while sediment depressions often harbor dense brines (Dando et al., 2000).

**Figure 2:**
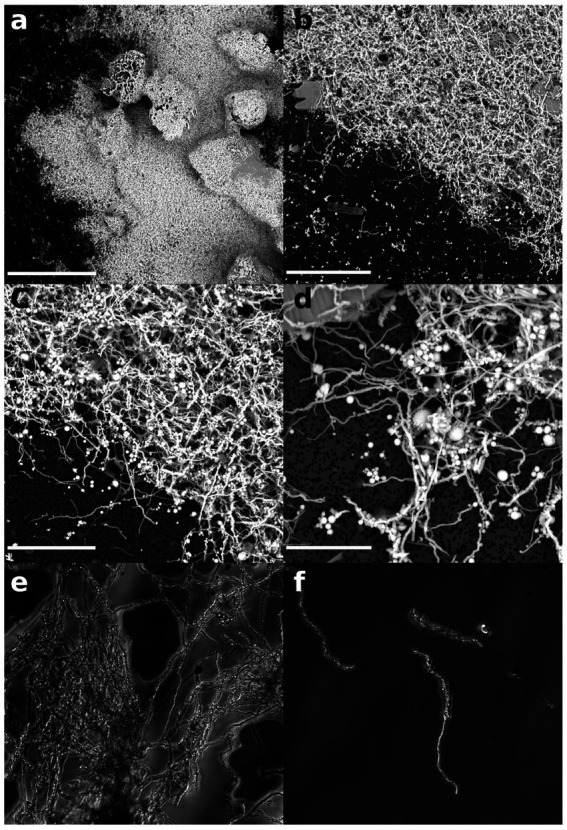
Scanning electron microscopy micrographs of Milos white microbial mats filtered onto a 0.22 μm filter. **(a)** A low-magnification overview of the microbial mats showing the high abundance of filamentous structures and the presence of large sand granules (scale bar 300 μm); **(b)** detail of a marginal section of the microbial mat structure showing the presence of long (20–50 μm) filaments and globular precipitates (scale bar 50 μm); **(c)** detail of the filamentous structures and globular precipitates. Each filaments is coated with a large number of small precipitates (scale bar 20 μm); **(d)** close up of the filamentous structures and globular precipitates (scale bar 10 μm); and **(e,f)** phase contrast micrograph of the filaments (1,000x). The globular precipitates are visible as bright spots under this light.

Within the extreme conditions found in the Milos hydrothermal systems, biofilms and microbial mats can attain thicknesses of several centimeters (Figure 2), representing the most abundant biomass structures (Hall-Stoodley et al., 2004). These biofilms offer homeostasis in fluctuating environments, potentially mitigating variations in pH, oxygen levels, exposure to ultraviolet radiation, and detoxifying heavy metals while concentrating nutrients. By confining cells in close proximity, biofilms facilitate inter- and intra-specific chemical signaling and nutrient exchange (Hall-Stoodley et al., 2004; Garnett and Matthews, 2012; Patwardhan et al., 2021). The presence of thick white microbial mats, which form at the interface of an oxygen-rich environment, such as in contact with both seawater and a H_2_S-rich environment, where hydrothermal fluids are discharged, is a prominent feature of hydrothermal systems on the seafloor (Sievert and Vetriani, 2012; Giovannelli et al., 2013; Yücel et al., 2013).

Despite their importance and visual prominence, the taxonomic and functional diversity of white mats in the Paleochori Bay has never been explored in response to hydrodynamics events. In this study, we combined 16S rRNA amplicon sequencing, from DNA and RNA, and metagenomic sequencing to investigate microbial community composition before, during, and after major storm events in Paleochori Bay. We also investigated mat structuring following hydrodynamic interactions with the sandy bottom. By integrating these approaches, we unravel the extent to which environmental perturbations shape microbial communities in SWHVs and provide new insights into the resilience and adaptability of biofilm-associated microbiota in fluctuating conditions. Our findings contribute to a broader understanding of the complex geobiological interactions governing hydrothermal vent ecosystems and highlight the importance of considering dynamic events when studying microbial diversity in geothermal ecosystems.

## 2 Materials and methods

### 2.1 Sites and sample collection

Samples were collected from May 21st to May 29th, 2012, from the shallow-water vents of Paleochori Bay in the island of Milos, Greece. The sampling site was characterized by shallow depth (9–13 meters) (Yücel et al., 2013) and the presence of thick microbial mats (Giovannelli et al., 2013; Puzenat et al., 2021; Le Moine Bauer et al., 2023), enabling close monitoring of the hydrodynamic disturbances impact on hydrothermal activity and microbial community (Figure 1). White mat samples used to assess the taxonomic and functional diversity were sampled during a period of calm sea state when the mats were mature and reached the thickness of a few centimeters. Samples for assessing the spatial changes in composition across small scales (i.e., at the scale of a single sand ripple) were acquired during the same period of time. Different coloured microbial mat samples were collected across sand ripples following the changes in the visual composition of the community from white, to yellow to brown. These changes occurred on sand ripples of 15–20 cm length from crest to crest. Samples used to analyze the microbial community composition after a major dynamic event were sampled before and after a 5 and 7 day period from a storm event with winds reaching up to 50 m s^−1^ (Yücel et al., 2013). All microbial mats samples were collected by SCUBA divers using sterile syringes and stored directly on the seafloor either in empty sealed hungate tubes or in hungate tubes pre-filled with RNAlater solution using a18G needle. Samples for molecular analysis were frozen 12 h after collection to allow for the RNAlater solution to stabilize the mats. Aliquots of the mats were preserved in 2% glutaraldehyde for microscopy analysis.

### 2.2 Scanning electron microscopy and phase contrast microscopy

Fragments of the microbial mats preserved in glutaraldehyde (2% final concentration) were recovered on a 0.22 μm pore-size Nucleopore polycarbonate filter, then frozen at −20 °C, mounted on a temperature controlled sample holder, and visualized at −25 °C on a Phenom ProX microscope (Phenom World). Images were acquired at maximum resolution using either a 10 kV or 15 kV acceleration voltage. Energy-dispersive X-ray spectroscopy (EDS) was performed at 15 kV acceleration voltage using the Phenom ProX EDS module. Microbial biofilms were also observed under optical microscopy using phase contrast at 1,000x magnification.

### 2.3 RNA and DNA extractions

Community DNA was extracted from RNAlater-fixed biomass using a modified phenol:chloroform protocol, following the method outlined by Giovannelli et al. (2013). Briefly, biomass aliquots (ca. 0.5 mL) were centrifuged to remove the RNAlater solution, followed by the resuspension of the pellet in extraction buffer (100 mM Tris–HCl, 100 mM EDTA, 1.5 M NaCl pH 8.0) supplemented with lysozyme (10 mg mL^−1^), and incubated at 37 °C for 30 min. Proteinase K (20 mg mL^−1^) was added, followed by another 30 min-long incubation at 37 °C. The sample was then treated with 20% SDS and agitated at 60 °C for 1 h. Precipitates and cell debris were removed by centrifugation (5 min at 14,000 × g) and the supernatant was collected and extracted with 1 volume of phenol:chloroform:isoamyl alcohol (25:24:1, pH 8.0), followed by one extraction with chloroform:isoamyl alcohol (24:1). DNA was then precipitated overnight with sodium acetate and isopropanol, washed with 70% ethanol, and resuspended in PCR grade water. DNA was visualized on 1% agarose gel stained with ethidium bromide and quantified on a NanoDrop 2000 spectrophotometer (Thermo Scientific). RNA extraction was carried out using a similar procedure, with acidic phenol used instead of phenol:chloroform:isoamyl alcohol, and the pellet was resuspended in diethylpyrocarbonate (DEPC)-treated water. RNA samples were then treated with DNAse I TURBO DNA-free kit (Ambion) following the manufacturer protocol. This DNase treated RNA was reverse transcribed into cDNA using the Invitrogen cDNA synthesis kit (Invitrogen, Carlsbad, CA, United States), according to the manufacturer’s specifications. Appropriate negative and no-RT control were carried out. Obtained cDNA was tested by PCR using 338F/517R bacterial primers targeting the 16S rRNA gene (Muyzer et al., 1993).

### 2.4 Pyrotag sequencing and analysis

Pyrotag amplicon sequencing was performed on the Roche 454 GS-FLX sequencer at MR DNA,^1^ using the cDNA as template and the bacterial primer 27F mod and 530R (Dowd et al., 2008). White microbial mat samples collected before (on the 21st May 2012), during (on the 26th May 2012) and after the storm event (the 28th May 2012), along with samples of different mats collected across the sand ripple (yellow, orange and green) were subjected to DNA extraction and 16S rRNA gene amplicon sequencing, concluding a total of six samples. Pyrotag amplicon sequencing was performed as specified above, by targeting the variable regions V1-V3 with the Roche 454 GS-FLX sequencing platform at MR DNA (see text footnote 1) (Dowd et al., 2008).

Generated raw 16S rRNA gene sequences were processed and analyzed using the QIIME 1.8 software package (Caporaso et al., 2010). Pyrosequencing noise was removed by employing the Denoiser 0.91 (Reeder and Knight, 2010) included in QIIME. Chimeric sequences were removed using UCHIME (Edgar et al., 2011), included in USEARCH (6.0.152). We applied UCHIME in *de novo* mode to cluster sequences, as this has been shown to outperform closed reference clustering approaches (Westcott and Schloss, 2015). The determination of Operational taxonomic unit (OTUs) was performed with the UCLUST algorithm (Edgar, 2010) at 97% similarity, and a representative set was selected employing the QIIME scripts “pick_otus.py” and “pick_rep_set.py.” Singleton were removed from the dataset. Taxonomic classification of selected reference sequences (OTUs) was performed by similarity searches using the Ribosomal Database Project naive Bayesian classifier, version 2.12 (Wang et al., 2007), included in QIIME with a cut off level of 0.80 on the assignment confidence. Therefore, all taxonomic affiliations used herein are based on those used at the time of the analysis. Individual OTUs represented by more than 10 sequences were searched against the EzTaxon database (Chun et al., 2007) in order to identify the closest cultured relative.

### 2.5 Metagenome sequencing and analysis

High quality community DNA extracted from the undisturbed microbial white microbial mat before the storm was submitted to MR DNA lab (see text footnote 1) for shotgun sequencing on the Roche 454 GS-FLX platform. Sequences obtained from the metagenomic shotgun sequencing were uploaded to MG-RAST webserver (Meyer et al., 2008), followed by a demultiplexing, quality and length filtering and dereplication steps. The obtained QC reads were analyzed using the built in MG-RAST pipeline and downloaded for downstream analysis. The raw fastq sequences were analyzed using webMGA (Wu et al., 2011) and Real Time Metagenomic (Edwards et al., n.d.) web servers. Sequences were screened for ribosomal RNA genes using RNAmmer (Lagesen et al., 2007) and Metaxa (Bengtsson et al., 2011). Obtained 16S rRNA sequences were clustered and classified using UCLUST and the RDP classifier as described above. Metaphlan2 (Truong et al., 2015) was used for taxonomic profiling of the entire metagenome. Quality checked reads downloaded from MG-RAST were assembled into contigs using the CLC genomic workbench. Gene calling was performed on the obtained contigs using prodigal (Hyatt et al., 2010). Obtained coding sequences were analyzed using NCBI blastp service (McGinnis and Madden, 2004) against the non-redundant database and the KEGG GostKOALA web service (Kanehisa and Goto, 2000). Given the high amount of reads assigned to viruses, the quality checked reads were uploaded to MetaVIR (Roux et al., 2011). All raw data is available through ENA Bioproject PRJEB88540.

To link taxonomic classification to functional potential, representative OTU sequences were analyzed using BLAST (McGinnis and Madden, 2004) against the NCBI non-redundant database, allowing for functional assignment based on sequence homology.

### 2.6 Fluid flow model generation

The effects of hydrodynamic forcing in sand ripples at the vicinity of the shallow-water hydrothermal systems were inferred by applying both the Lattice Boltzmann Method (LBM) and Finite Volume Method (FVM) to model the interaction between the tide and shear imposed fluid flow, thermal advection and an obstacle represented by a sand ripple. The FVM was adopted to solve the flow model, based on the Navier–Stokes equations with a momentum sink term to represent soil porosity. Specifically, a colocated unstructured FVM strategy was used (Greenshields and Weller, 2022). Furthermore, all diffusive terms and pressure gradients were approximated with second-order accuracy, while the convective terms were discretized using a linear-upwind approach. The aforementioned solution strategy was implemented within the well-known object-oriented OpenFOAM open-source library.

We performed lattice Boltzmann modeling (LBM) simulations to examine water flow around and through a simplified ripple geometry. Flow within the computational domain was driven by imposing a constant horizontal velocity at the upper boundary, simulating shear flow conditions. Both the ripple structure and the underlying sand bed were represented as porous media, permitting fluid penetration to a limited extent. In addition, a vertical temperature gradient was imposed throughout the domain. This gradient was deliberately set below the threshold required to induce natural convection, thereby ensuring that heat transport within the system occurred exclusively via advection associated with the imposed horizontal flow. Model parameters, including upper boundary flow velocity, sediment porosity, and the magnitude of the temperature gradient, were selected based on typical environmental conditions observed in sediment-water interaction scenarios (Precht and Huettel, 2004).

The code for implementing this LBM was written in C with MPI routines for parallelization. It can be found at the following github repository: https://github.com/giovannellilab/Milos_white_mats.

## 3 Results

### 3.1 Microbial mat structure and microscopic observations

A close association between the curved non-branching filaments and the small bright granules emerged from phase contrast observations of the microbial mat (Figure 2). Granules with diameters below 2 μm appeared to be attached to the outer surface of the filaments. The white microbial mat structure did not present classic evidence of well structured exopolysaccharides, but was composed of a network of loosely packed microbial filaments and mineral precipitates. EDS analysis of the larger granules revealed the presence of dominant sulfur peaks, suggesting the presence of sulfur-rich mineral phases, possibly including oxidized sulfur species and elemental sulfur precipitates. We also identified additional minerals embedded in the mat filaments, such as manganese oxide and arsenic sulphides. Individual cells shaped like elongated, curved rods and measuring 1.5–2 μm in length were visible under phase contrast, though they were not consistently present across all samples. The samples included a variety of microbial morphologies, such as vibroids, cocci, and rods. Filaments were the most abundant morphology observed. The granules were bright under phase contrast (Douglas, 2000; Kümpel et al., 2023).

### 3.2 Taxonomic and functional diversity of the white microbial mat community

**Figure 3:**
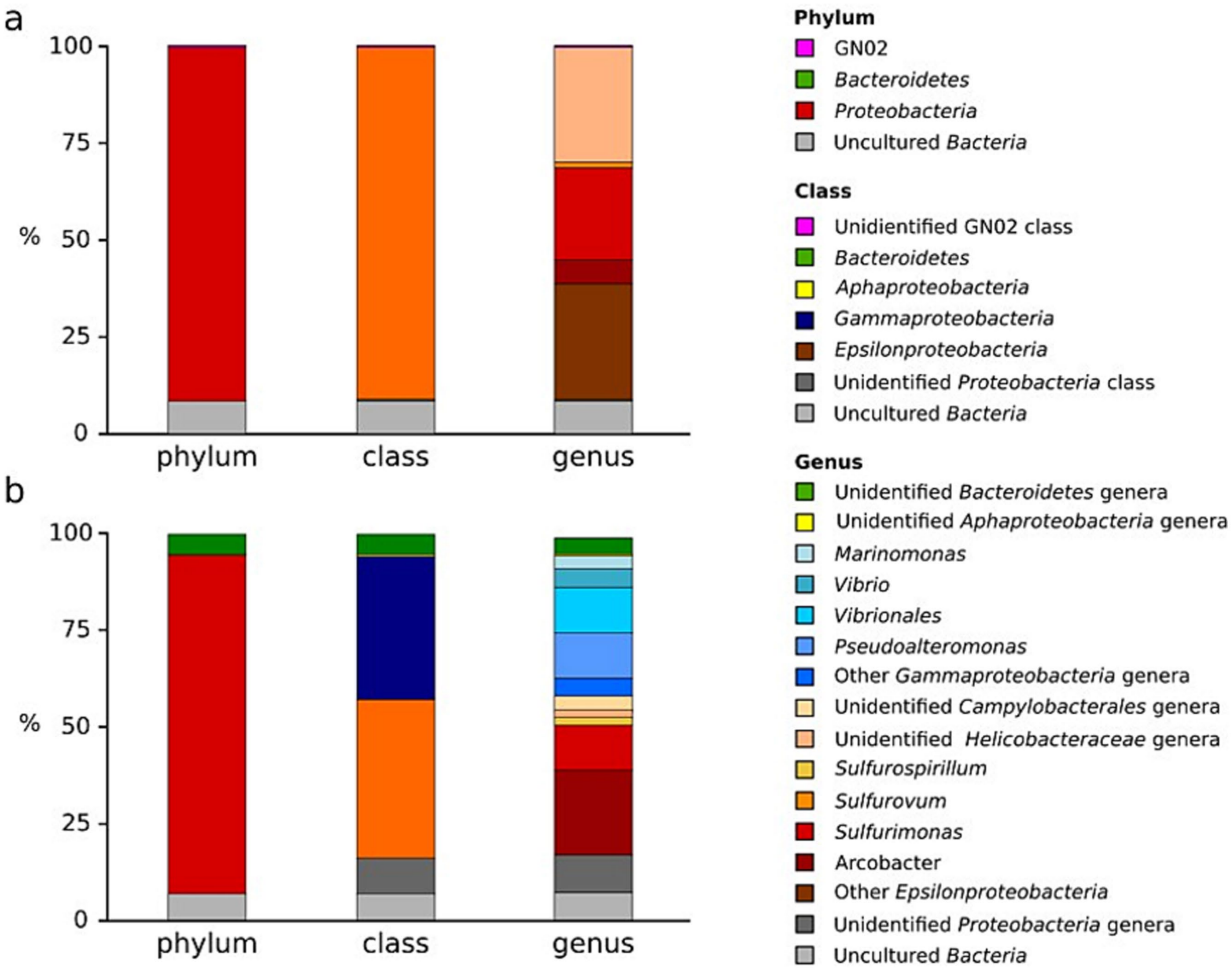
Comparison of the prokaryotic diversity in the single sample from the undisturbed microbial white mat before the storm event estimated using **(a)** the cDNA 16S rRNA pyrotags (active community) and **(b)** DNA (16S rRNA extracted from the metagenome). Taxonomic affiliation of the sequences is presented at the phylum, class and genus level.

The diversity and activity of the resident microbial community were investigated by analyzing and comparing the V1-V3 variable regions of the 16S rRNA gene transcripts, thus representing the actively transcribing community, with genes encoding for the 5S, 16S, and 23S rRNA extracted from the shotgun metagenomic sequencing (Figure 3). A total of 4,294 reads from the 16S rRNA gene transcripts sequencing passed quality control steps and were clustered in 804 unique OTUs at 97% similarity. The library is dominated by sequences belonging to the Proteobacteria (91%—all percentages refer to relative abundances), with the majority belonging to the Epsilonproteobacteria class (90.6%, now classified as Campylobacterota), and a small fraction not assigned to any known Proteobacteria class (0.4%; Figure 3a). The majority of the remaining sequences (8.5%) was not classified in any known bacterial phyla, while a very small fraction (0.5%) was classified into the candidate division GN02. Within the Epsilonproteobacteria, 23.8% was classified as belonging to the *Sulfurimonas* genus, 6.1% to *Arcobacter* genus, and 1.3% to the *Sulfurovum* genus. The remaining sequences within the Epsilonproteobacteria class were evenly classified as unidentified *Helicobacteraceae* (29.6%), and unidentified Epsilonproteobacteria (29.8%). A search of all the OTUs (each represented by more than 10 sequences) against the EzTaxon database revealed very low similarities (between 89 and 93%) against cultured strains, even for sequences for which the Ribosomal Database Project (RDP) classifier returned high confidence of assignment to specific genera.

A total of 559 partial 16S rRNA genes were recovered from the metagenomic library. The sequences were dominated by the Proteobacteria phylum (87.9% of the 16S rRNA sequences), followed by the Bacteroidetes (5.3%; Figure 3b). The remaining 16S rRNA sequences were represented by uncultured bacteria (6.9%). At the class level the Proteobacteria sequences were evenly assigned to the Epsilon- and Gammaproteobacteria class (41 and 36.9%, respectively), followed by unidentified Proteobacteria (9.4%), uncultured Bacteria (6.9%) and a small percentage of sequences assigned to the Alphaproteobacteria class (0.5%). The sequences belonging to the Epsilonproteobacteria were further assigned to the genera *Arcobacter* (21.9%), *Sulfurimonas* (11.6%), and *Sulfurospirillum* (2.1%), with the remaining sequences classified as either unidentified *Helicobacteraceae* (2.2%) or unidentified *Campylobacterales*. The sequences belonging to the Gammaproteobacteria were dominated by sequences closely related to the genera *Pseudoalteromonas* (11.8%), *Vibrio* (4.7%), *Marinomonas* (3.4%) and other unidentified *Vibrionales* (2.6%). The sequences related to the Alphaproteobacteria class of the Proteobacteria and the Bacteroidetes were not identified at the class or genus level.

**Figure 4:**
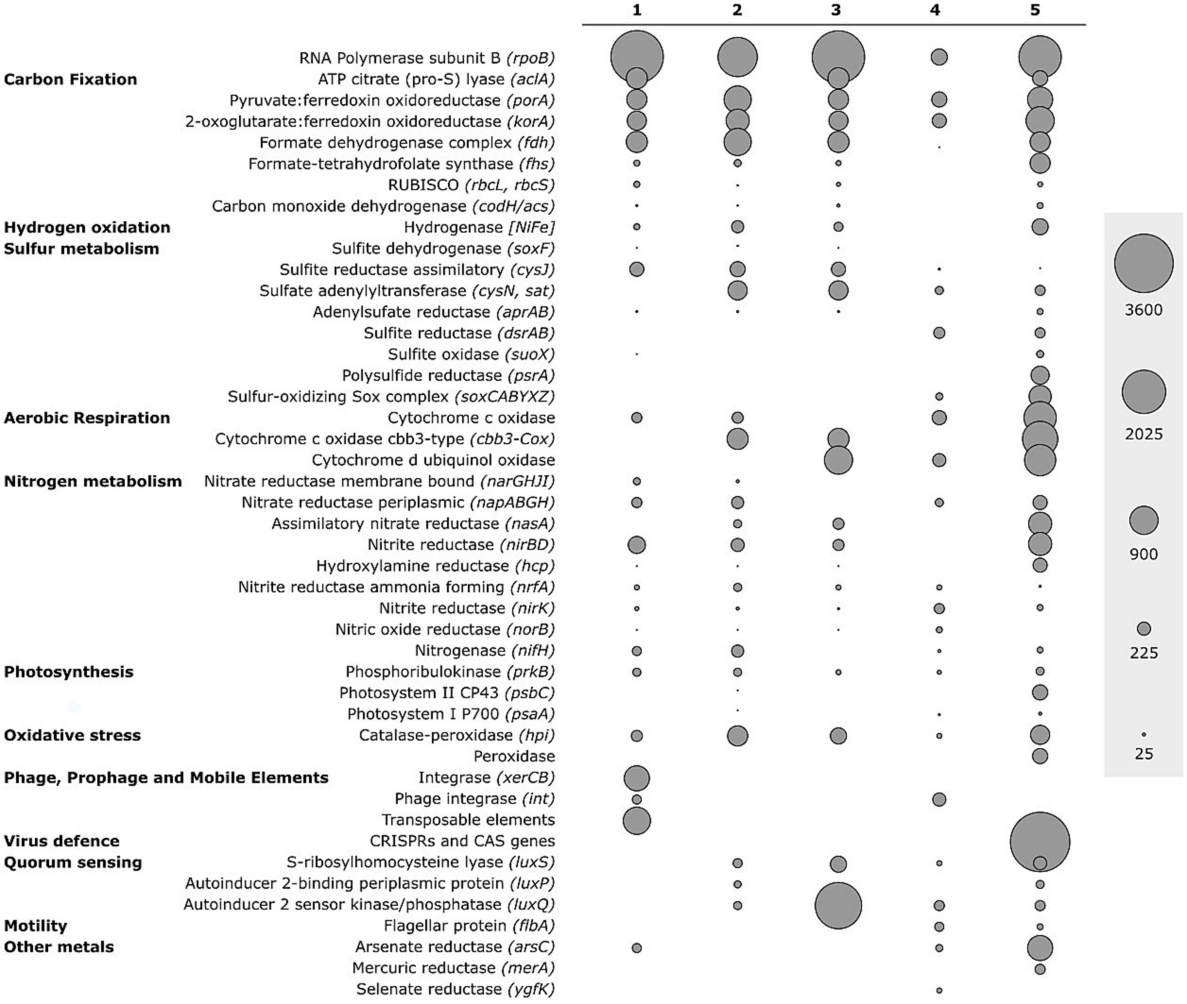
Abundance of reads assigned to key functional genes in the microbial mat metagenome using different approaches: (1) genes assigned to functions in the Kyoto Encyclopedia of Genes and Genomes (KEGG); (2) genes assigned to KEGG KO numbers using MG-RAST; (3) genes assigned to Enzyme Commission numbers using MG-RAST; (4) genes assigned to functions against the NCBI Protein Cluster database using WebMGA; (5) genes assigned to the RAST Subsystem database using Realtime MG. Raw gene counts are shown in Supplementary material.

Metagenomic shotgun sequencing of the white microbial mat resulted in 264,257,917 bp in 1,284,013 reads. These were assembled in a total of 3,988 contigs (N50 = 3,407 bp, total contigs length of 7,796,078 bp). Functional assignments made through the use of Kegg Orthologues (KO) and Enzyme Commission numbers (EC) on the MG-RAST database resulted in a diverse number of assigned reads (259,784 and 130,269 counts, respectively; Figure 4). By contrast, functional assignment using NCBI Protein Cluster through WebMGA and the RAST Subsystem database through Realtime MG yielded 149,920 and 3,205,896 assigned reads, respectively (Supplementary Table 1).

#### 3.2.1 Carbon and energy metabolism

The high abundance of sequences assigned to genes encoding for ATP citrate (*Pro-S*) lyase (*aclA*; up to 476 reads), for 2-oxoglutarate:ferredoxin oxidoreductase (*korA*; up to 814 reads) and for pyruvate:ferredoxin oxidoreductase (*porA*; up to 788 reads), key genes for the rTCA cycle, suggests that carbon fixation through this pathway was highly represented in the white mat metagenome (Hügler et al., 2005; Fuchs, 2011). Likewise, the identification of sequences assigned to the bifunctional carbon monoxide dehydrogenase/acetyl-CoA synthase gene (*codH/acs*; up to 43 reads), and to the formate dehydrogenase complex (*fdh*; up to 788 reads) hint to the relevance of the Wood–Ljungdahl pathway, also known as the reductive acetyl coenzyme A pathway.

The high number of sequences assigned to genes encoding for the SOX (sulfur oxidation) complex (up to 550 reads), responsible for the oxidation thiosulfate and sulfide, suggests that all major prokaryotic sulfur oxidation pathways typical of geothermal environments were widespread in the white microbial mat community (Ghosh et al., 2009; Chen et al., 2022). All subunits of the SOX gene complex are detected in our dataset, suggesting the presence of a complete thiosulfate oxidation pathway in the white mat microbial community. In addition, genes associated with both assimilatory and dissimilatory sulfate reduction pathways are present, indicating that the community held the potential for multiple sulfur transformations (Figure 4) (Weissgerber et al., 2013; Dahl, 2017).

We retrieved a consistent set of nitrogen cycle-related sequences across all five annotation databases. Specifically, we identified sequences assigned to gene *nifH* (up to 178 reads) encoding one subunit of nitrogenase, the enzyme complex responsible for biological nitrogen fixation under anaerobic or microaerobic conditions (Mehta et al., 2003; Chen et al., 2023). In addition, sequences assigned to genes involved in dissimilatory nitrate reduction and denitrification pathways were recovered, including *narGHJI* (up to 62 reads), *napA* (up to 215 reads), *nirB* (up to 565 reads), *nirK* (up to 112 reads), *nrfA* (up to 75 reads), and *norB* (up to 47 reads), indicating the potential for both respiratory and dissimilatory nitrate/nitrite reduction mechanisms (Pérez-Rodríguez et al., 2013; Reyes et al., 2017; Patwardhan et al., 2021, 2023). Sequences assigned to gene *nasA* (up to 589 reads), encoding the nitrate reductase involved in assimilatory nitrate reduction, were also present, supporting the role of nitrate as a nutrient source in this community.

Oxidative phosphorylation in the white mat microbial community appeared to be facilitated by the presence of multiple terminal oxidase complexes and oxidative stress defense enzymes. Of significance is the presence of the cytochrome c oxidase cbb3-type gene (*cbb3-Cox*; up to 506 reads). Furthermore, our analysis revealed the presence of sequences assigned to catalase-peroxidase gene (*hpi*, up to 435 reads) and bacterial cytochrome c peroxidase gene (*ccp1;* 249 reads).

#### 3.2.2 Additional functions: mobile elements, quorum sensing and motility, and heavy metal detoxification

The analysis highlighted the presence of sequences assigned to phage integrase-related genes (*xerC*; 692 reads), which encode proteins mediating unidirectional site-specific recombination between the phage attachment site (attP) and the bacterial attachment site (attB) on two DNA recognition sequences (Groth and Calos, 2004).

Our read-based analysis revealed the presence of genes encoding for autoinducer ligands (*luxS*), which are released by bacteria to monitor cell density. The presence of the gene *flbA* encoding for a flagellar protein, further highlights motility related to quorum sensing.

Our sample shows the presence of sequences assigned to key genes involved in heavy metals detoxification pathways, such as arsenate reductase (*arsC*; up to 687 reads), mercury reductase (*merA*; up to 130 reads) and selenate reductase (*yfgK*; up to 32 reads).

#### 3.2.3 Taxonomic affiliation of the functional genes

**Figure 5:**
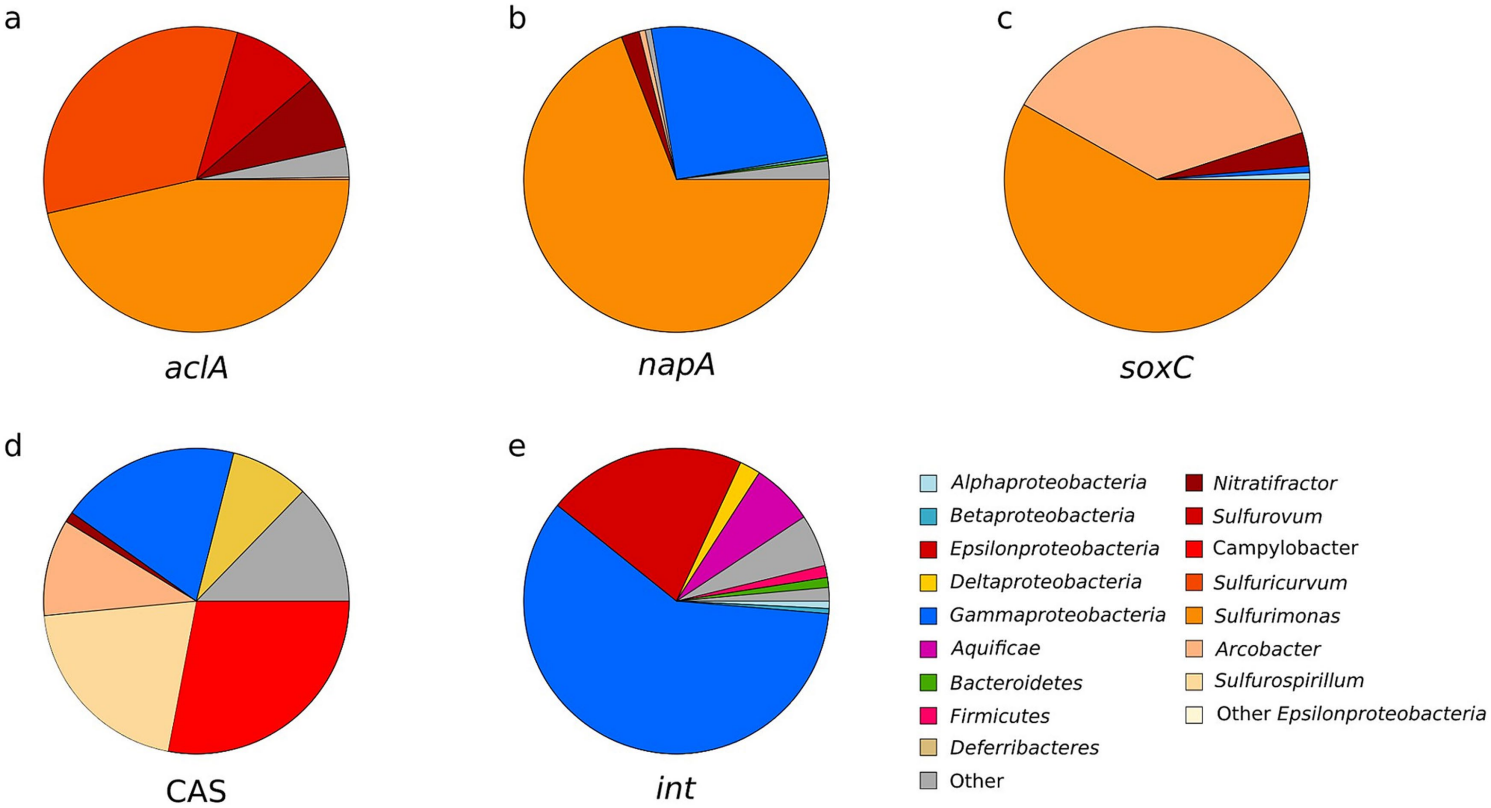
Taxonomic affiliation and relative abundance of key selected genes recovered in the metagenome. Diversity of recovered ATP citrate lyase alpha subunit gene *aclA* **(a)**; periplasmic nitrate reductase catalytic subunit gene *napA* **(b)**; thiosulfate oxidation protein coding gene *soxC* **(c)**; CRISPR associated sequences gene diversity **(d)**; and diversity of transposon and phage integrase gene *int* **(e)**. In all plots the diversity of sequences associated with the Epsilonproteobacteria is shown at the genus level, except in plot **(e)**, where the sequences are combined at the class level.

To better understand the diversity of the microorganisms involved in diverse biogeochemical pathways, we taxonomically annotated the main functional genes of each major element cycle, showing interesting relationships between microbial diversity and functions in the SWHV white mat (Figure 5). This analysis revealed that the genes involved in the rTCA cycle, dissimilatory nitrate reduction, denitrification, thiosulfate oxidation and *CAS* genes were assigned to Epsilonproteobacteria. The relative abundance of *aclA* is divided among *Sulfurimonas* (46.4%), *Sulfuricurvum* (32.9%), *Sulfurovum* (9.3%) and other related genera (11.3%). The taxonomic assignment of the functional genes was consistent with the microbial composition obtained with the 16S rRNA transcripts (Figure 5). Thiosulfate oxidation gene (*soxC*) sequences were assigned to *Sulfurimonas* (58.2%), *Arcobacter* (36.9%) and *Nitratifractor* (3.5%) taxonomic groups. Dissimilatory nitrate reduction and denitrification (*napA*) showed a similar distribution compared to *soxC*, with high dominance of *Sulfurimonas* (69.1%), and differed from the latter as *napA* is also consistently assigned to Gammaproteobacteria (*Vibrio* 16.1% and *Alteromonadales* 4.8%). In relative abundance, CAS genes were dominated by the genera *Campylobacter* (28%), *Sulfurospirillum* (20.5%) and *Arcobacter* (10.2%). The diversity data about transposon and phage integrase gene (*int*) showed, contrary to the others genes, a dominance of Gammaproteobacteria (59.5% of taxonomically annotated genes), followed by Epsilonproteobacteria (21.1%).

### 3.3 Changes in microbial community composition before and after a storm event

**Figure 6:**
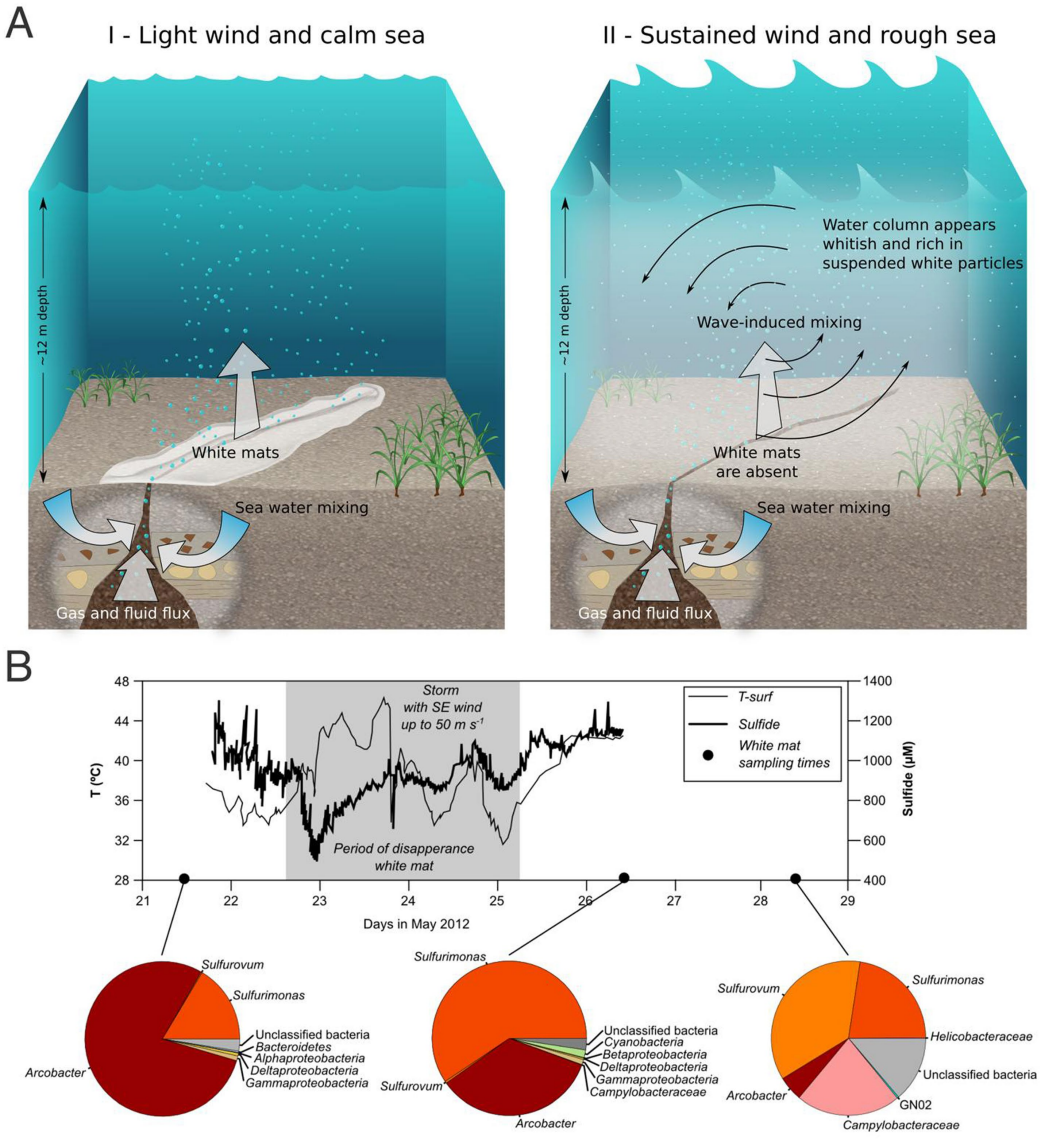
**(A)** Reproduction of establishment and disruption of white mat precipitate in response to hydrothermal fluid emissions. Dynamics of the site under light wind and calm sea (I), during which the SCUBA Divers observed the deposition of the white precipitate and fluid emissions unaffected by currents. Dynamics of the site under sustained wind and rough sea (II), during which the SCUBA Divers observed the disruption of the white mat precipitate and its related resuspension, resulting in a whitish water column rich in suspended material. **(B)** Time series of *in situ* sensor measurements coupled with temporal changes in the microbial community based on cDNA 16S rRNA amplicons. The gray area indicates the approximate timing of the high wind and wave conditions and the corresponding mat-free period. In this period, maximum wind speeds approaching 50 m s^−1^ were recorded on May 23rd. Temperature close to the surface of the sediment (10 mm depth, 0.5 m away from the vent center) is represented by a thin black line. The total dissolved sulfide time series at 10 mm below the sediment–water interface is indicated by a bold line. Sampling to understand the community composition and its variation over time are conducted, respectively, one day before disturbance (pre-storm conditions), one day after it (post-storm conditions—mats re-establishment) and three days after it (post-storm conditions—mats development).

Field observations and microscopy analysis suggest that the microbial mat is loosely attached to the sandy seafloor, likely as a result of benthic deposition due to the presence of a high percentage of minerals attached to the microbial filaments and dispersed in the matrix. Rapid movements of the SCUBA divers or increased lateral fluid flow near the seafloor due to currents and waves can easily resuspend the mats that dissolve in seawater creating a milky resuspension (Figure 6). Hydrodynamic stress and water column mixing is minimal during periods of calm sea conditions, allowing the establishment of the white microbial mats around the outflow zones of the shallow vent, and leading to the creation of stable white patches that might grow to several cm thickness (Figures 2, 6A). Our data show that immediately prior to a storm, the white mat community was dominated by *Arcobacter* (78.3%), followed by a lower percentage of *Sulfurimonas* and unclassified bacteria (16.3 and 2.5%, respectively). During the storm events, the white mat disappeared due to blowing of South-East (SE) wind, characterized by velocities up to 50 m s^−1^. The storm also determined a shift in the sulfide and temperature regime of the vent (Figure 6B; Yücel et al., 2013). The white mats reappeared as thin white layers one day after the storm. The samples taken one day after the disturbance event showed a shift in community toward dominance of *Sulfurimonas* (59.1%), with a lower abundance of *Arcobacter* (34.4%). Notably, *Cyanobacteria* relative abundance increased after the storm event (1.6% compared to pre-storm abundance of 0.3%). Three days after the disturbance event, there was a further shift to a community dominated by *Sulfurovum* (36.2%) and *Sulfurimonas* (22.5%), with an additional decrease of *Arcobacter* (5.2%), disappearance of *Cyanobacteria,* and higher abundance of *Campylobacteraceae* (22.0%) and unclassified bacteria (13.8%) (Figure 6).

### 3.4 Fluid flow across the sandy bottom

The interaction of buoyancy-driven fluid flow discharge with ripple topography modifies the direction and mixing of vent fluids. As hot, buoyant fluid convects upward from subsurface conduits, it encounters lateral flow from bottom currents driven by regional circulation and surface wind forcing. When this convective upwelling intersects ripple crests and troughs, it generates heterogeneous flow regimes, including zones of focused upwelling, lateral shear, and recirculation eddies. It is very important to put evidence that ripple shape induces flow acceleration over the ripple crest and a consequent pressure drop in accordance with the Euler equation in streamlines coordinates (Supplementary Figure S1A). The deriving pressure field generates a vertical stream (Supplementary Figure S1B); on the contrary high pressure zones in the ripple troughs inhibit discharge or can produce a lateral flow. Moreover, the aforementioned pressure difference produces a mixing layer between the involved fluids and the resulting effect is chemical species gradients spatial variability.

Actually, ripple shape produces a particular flow field with a strong heterogeneity in fluid chemical composition, temperature, and redox conditions over centimeter scales. Crest regions may be more exposed to oxygenated seawater, while troughs trap reducing hydrothermal fluids, creating sharp horizontal and vertical gradients in electron donors and acceptors. As a result, the relative contribution of hydrothermal versus seawater inputs varies dramatically across the ripple, directly shaping the chemistry of porewater and the structure of microbial mats growing on the surface. These microscale variations help explain the spatial patchiness observed in microbial community structure and function described in Section 3.4.

We conducted LBM studies of the flow around and through a simplified ripple shape. The driving force of the flow was an assumed constant velocity (horizontal shear flow) upper boundary (the upper layer of grid cells). The ripple and sand bed are assumed porous so there is a modest flow through both. We see in Supplementary Figure S1 that there is boundary layer separation due to the ripple, and a subtle recirculation eddy on the lee side of the ripple. We impose a vertical temperature gradient, as shown in the lower panel of Supplementary Figure S1C. However, this temperature gradient is not sufficient to drive natural convection, hence advection of heat occurs only due to the flow past the ripple (the eddy described above).

### 3.5 Spatial variability in microbial community composition across a sand ripple

**Figure 7:**
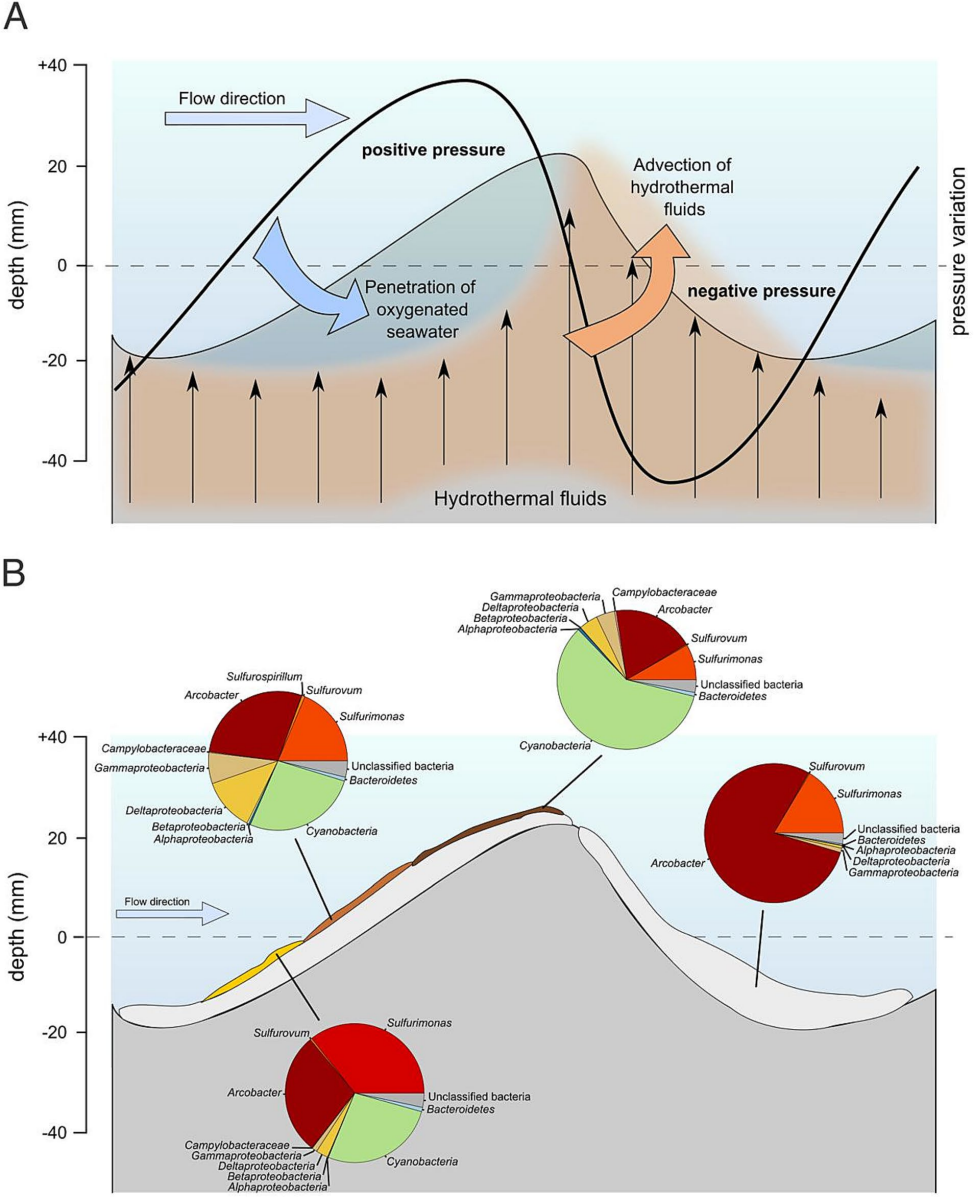
**(A)** Proposed model depicting the dynamics of water flow surrounding the vent structure and its interaction with vent fluid emissions. The area under consideration extends to a depth of 20–40 mm within the sediments. The outflow of hydrothermal fluids creates a void, generating negative pressure, which in turn establishes a positive pressure gradient from top to bottom inside the sand ripple. The flow direction of water currents facilitates the thinning of the benthic boundary layer over the gentle slope of the ripples, enabling increased oxygen penetration into the sediments and facilitating the growth of associated microbial mats. **(B)** Microbial community gradient established by different degree of oxygenation and its comparison with a community of the opposite side of the ripple (steep one) rich in H_2_S. Differences regarding the composition in phototrophic players are created by different oxygenation of the geo-biological system. The light-grey layer beneath the colored microbial mats (yellow, Siena red, brown) represents the underlying white microbial mat that serves as a basal layer upon which the overlying colored microbial communities develop.

Near the venting location in the shallow-water hydrothermal systems in Paleochori Bay we observed microbial mats of different colours (e.g., white, yellow, light brown and brown mats) in close proximity following single sand ripples. Analysis performed on these differently coloured mats provide insights into the short-scale spatial variability of microbial communities in these systems (Figure 7). White colored mats were consistently present in the sand ripple valleys, and yellow and brown colors appeared to be preferentially located on the sand ripple flank and crest. This distribution suggested a physical control over the microbial composition of the mats.

White mat composition has been described in section 3.2 (Figures 3, 7). The yellow mat community was dominated by *Proteobacteria* (68.2%). At the class level, *Epsilonproteobacteria* dominated the community (64.0%), with a high abundance of *Arcobacter* (28.2%). In contrast, the light brown mat showed higher variability at the class level, with a similar abundance of *Proteobacteria* (66.6%), but a reduced proportion of *Epsilonproteobacteria* (46.7%), showing differences with the white mat due to a higher percentage of *Deltaproteobacteria* and *Gammaproteobacteria* (12.0% and 7.0%, respectively). The brown mat, compared to the other types, showed increased differences, with a lower proportion of *Proteobacteria* (36.4%) represented mainly by sequences belonging to the *Epsilonproteobacteria* class (27.2% of the total). The genus *Arcobacter* was less represented when compared to the other types of mat (18.5%). The *Cyanobacteria* abundance varied across the sand ripple surface, increasing from the yellow mat to the brown mat (26.6% in the yellow mat; 26.0% in the light brown mat; 57.6% in brown mat). Moreover, we observed a decreasing gradient of *Sulfurimonas* presence along the sand ripple surface, which lowered towards the brown mat (35.3% in the yellow mat; 18.1% in the light brown mat; 8.1% in the brown mat).

## 4 Discussion

### 4.1 White mat diversity and composition

Shallow-water hydrothermal vents are ubiquitous systems in coastal marine environments characterized by volcanic activity (Price and Giovannelli, 2017). The primary productivity in these systems is driven both by photosynthesis and chemosynthesis carried out by diverse microbial groups (Tarasov, 2006; Dando, 2010). The relative contribution of the two is controlled by complex physical and chemical interactions between the advecting fluids, the seafloor features and seawater column processes (Price and Giovannelli, 2017; Giovannelli and Price, 2019). Biofilm and microbial mats represent an important strategy for microorganisms to colonize these dynamic ecosystems and constitute the base of the food web sustaining local diversity and possibly exporting carbon and nutrients to neighboring ecosystems (O’Brien et al., 2015; Sciutteri et al., 2022). Milos shallow-water hydrothermal vents are dominated by white microbial mats loosely attached to the sandy bottom in areas of hydrothermal fluid discharge (Giovannelli et al., 2013; Price et al., 2013; Yücel et al., 2013). The mat exhibits a filamentous structure intertwined with sulfur-rich granules (Sievert et al., 1999), either attached to filaments or dispersed within the matrix. The composition of the biofilm communities shows similarities to those found in other shallow hydrothermal vent systems worldwide (Kamenev et al., 1993; Dando et al., 1999; Wenzhöfer et al., 2000; Cardigos et al., 2005; Lentini et al., 2014). Elemental analysis (EDS) of the sulfur granules confirms their composition (Figure 2). The detection of sulfur-oxidizing bacteria, particularly *Epsilonproteobacteria,* in the active fraction of the community, further supports their possible role in the biological mediated precipitation of sulfur granules. Members of this class, especially the genus *Arcobacter*, have been implicated in filamentous sulfur structures in similar environments (Sievert et al., 1999, 2007; Wirsen et al., 2002). This functional role is further corroborated by the abundance of *sox* gene clusters identified in the metagenomic dataset, which include genes such as *soxB,* key components of the sulfur oxidation pathway known to lead to the accumulation of elemental sulfur as an intermediate (Friedrich et al., 2005; see below section 4.2). Altogether, given that both biotic and abiotic processes can generate such structures, the strong microbial activity observed in this site (Giovannelli et al., 2013) likely plays a key role in their formation.

The higher abundance of *Sulfurimonas* spp. in the 16S rRNA transcriptome (Figure 3a) could be related to their metabolic flexibility, such as the utilization of a wide range of electron donors of geothermal origin, including sulfide, sulfur, thiosulfate, sulfite, and hydrogen, while employing nitrate, nitrite, or oxygen as electron acceptors. *Sulfurimonas* spp. have been shown to use CO_2_ as their primary carbon source, though some species have also been reported to metabolize acetate (Giovannelli et al., 2013; Labrenz et al., 2013). Similarly, *Arcobacter* and *Sulfurovum* genera exhibit wide metabolic flexibility in the usage of several electron donors and acceptors of hydrothermal origin (Xie et al., 2021). Previous studies have shown the *Arcobacter* spp. implication in sulfur filaments formation within the white mats of Milos shallow vents (Sievert et al., 2007). Additionally, members belonging to *Sulfurimonas* have been widely described in other shallow water and deep-sea hydrothermal systems (Wang et al., 2017, 2021). The presence of an active community with distinct members that use multiple species of sulfur as electron donors hints at their ecological adaptation to shallow water hydrothermal systems and at their crucial roles in the sulfur cycle therein. Notably, no sequences associated with the Gammaproteobacteria genus *Galenea* (Giovannelli et al., 2012), a chemolithoautotrophic thiosulfate oxidizing organism originally isolated from Milos shallow vents, have been found in the libraries. This could be due to its low abundance at the time of sampling, or to biases in the annotation database used.

In contrast to the active community revealed by 16S rRNA transcripts (Figure 3a), the total resident community captured by 16S rRNA genes in the metagenomes (Figure 3b) shows a higher abundance of *Gammaproteobacteria* and *Bacteroidetes*. Among *Gammaproteobacteria*, the genus *Pseudoalteromonas* is notably dominant, aligning with its known roles in organic matter degradation and metabolite production in marine hydrothermal systems (Sievert et al., 1999; Patwardhan et al., 2021; Lu et al., 2020; Price and Giovannelli, 2017; Holmström and Kjelleberg, 1999). Other notable genera include *Vibrio*, and *Marinomonas*, both associated with nutrient cycling and adaptability to hydrothermal fluid interactions (Huang et al., 2020; Huber et al., 2003). This comparison revealed that the active community represents only a fraction of the total resident microbial groups. The active taxonomic groups do correspond to a substantial proportion of the total community, while the low-abundance genera are metabolically inactive, quiescent.

The observed groups are well known members of the water column; their detection in the gene (but not transcript) dataset suggests they are delivered to the white mats by the short-scale circulation patterns reported by Yücel et al. (2013). This mechanism works in a way that microbial taxa present and active in the water column are trapped by short scale hydrothermal recharges in the white mats that act as a mechanical filter. This brings into the active mats, dominated by Epsilonproteobacteria, new trophic and genetic resources, potentially sustaining the heterotrophic community and favouring lateral gene transfer between the water column genetic pool and the hydrothermally sustained active white mat community. This hypothesis is supported by the presence of integrase and CAS associated genes in the white mat metagenome that are of gammaproteobacterial origin (Figure 5).

Viral analysis revealed the predominance of dsDNA viruses primarily from the order *Caudovirales*, including families *Siphoviridae*, *Myoviridae*, and *Podoviridae* (Supplementary Figure S2). These phages likely play a role in microbial population control and horizontal gene transfer, potentially influencing community structure and metabolic capacity (Howard-Varona et al., 2020).

### 4.2 White mat contribute to carbon, sulfur and nitrogen cycling in Milos shallow vents

The microbial communities inhabiting shallow-water hydrothermal vents off Milos, Greece, exhibit a diverse functional profile. The high abundance of genes involved in the rTCA cycle suggests this might be the primary pathway for carbon fixation, consistent with the dominance of Epsilonproteobacteria, particularly *Sulfurimonas*, *Sulfuricurvum*, *Sulfurovum* which rely on this cycle (Hügler and Sievert, 2011; Wang et al., 2017). This metabolic strategy, common in both shallow and deep-sea hydrothermal vents, likely reflects adaptation to CO_2_-rich conditions from magmatic degassing and the microaerophilic to anaerobic conditions (Hügler and Sievert, 2011; Giovannelli et al., 2013; Wang et al., 2017). Epsilonproteobacteria have been previously identified as major chemoautotrophs in similar systems, supporting their role as primary producers (Tarasov et al., 2005; Giovannelli et al., 2013; Wang et al., 2015).

Sulfur metabolism is well-represented with a strong presence of *sox* genes associated with sulfur oxidizing bacteria, particularly Epsilonproteobacteria (Dahl, 2017; Patwardhan et al., 2021). Thermodynamic calculations of the Milos shallow water vents systems suggest thiosulfate/sulfide oxidation through the SOX pathway as highly energy-efficient for chemolithotrophs (Lu et al., 2020). Genes such as *soxCD* and polysulfide reductase indicate a mix of sulfur-storing and non-storing microorganisms, supporting the biogenic origin of sulfur globules seen microscopically (Dahl, 2017). The detection of genes involved in both sulfur oxidation (*sox* genes) and those typically linked to sulfate reduction (*sat*, *aprAB*, *dsrAB*) highlights the metabolic versatility of the community in adapting to sulfur-rich conditions. While these genes are commonly associated with dissimilatory sulfate-reducing pathways (Pereira et al., 2011; Müller et al., 2015), their potential bidirectional function, including use in oxidative sulfur metabolism (Dahl, 2017), prevents definitive assignment of functional direction. Nonetheless, their co-occurrence suggests the community harbors both oxidative and reductive sulfur metabolic potential, consistent with dynamic redox conditions at hydrothermal vents.

Nitrate reduction is another prominent pathway. The *napA* gene, involved in nitrate reduction, was the most abundant and mainly attributed to *Sulfurimonas*. Its presence is widespread in Epsilonproteobacteria from marine hydrothermal systems, and the enzyme’s high affinity for nitrate may be related to the adaptation to environments with low nitrate concentrations, such as hydrothermal vents (Vetriani et al., 2014) and other nitrate-limited habitats (Han and Perner, 2015). This aligns with previous studies that have linked the process of denitrification to *Sulfurimonas denitrificans*. This process plays a critical role in balancing the nitrogen and carbon cycles in hydrothermal vent ecosystems by regulating nitrogen availability and supporting microbial metabolism (Grote et al., 2012; Sievert and Vetriani, 2012). Additional genes for dissimilatory nitrate reduction, assimilatory nitrate reduction, and nitrogen fixation suggest flexible nitrogen cycling strategies with oxygen-limited zones (Vetriani et al., 2014; Sievert and Vetriani, 2012; Tang et al., 2013). Dissimilatory nitrate reduction and denitrification (n*apA*) have mainly been attributed to the genus *Sulfurimonas* (69.1%) in our reads, but these pathways differ as to the association to Gammaproteobacteria (*Vibrio* 16.1% and *Alteromonadales* 4.8%, respectively). These data indicate that a significant fraction of the chemolithoautotrophic production at shallow-water vents could be coupled to nitrate reduction.

Aerobic and microaerophilic oxygen respiration appears to be supported by cytochrome c oxidase (cbb_3_-COX), which can be coupled with the oxidation of H_2_S and thiosulfate found in white mat areas (Yücel et al., 2013). In these environments, microaerophilic bacteria thrive at the interface where oxygen (O₂) and hydrogen sulfide (H₂S) gradients meet (Yücel et al., 2013). The cbb3-type cytochrome c oxidase is particularly well adapted to these conditions as it has a high affinity for oxygen, allowing bacteria to efficiently respire in low-oxygen (microaerobic) zones while simultaneously oxidizing sulfide (Smirnov et al., 2009). Cytochrome c oxidase affiliated with the *Sulfurimonas* genus (Labrenz et al., 2013), and other Epsilonproteobacteria (Campbell et al., 2006) support its functional role as terminal enzymes in the bacterial respiratory chain (bd complex) (Borisov et al., 2011, 2021). The detection of cytochrome c (Cyt-c) further indicates a complete electron transport chain, acting as a soluble electron carrier that facilitates electron flow between dehydrogenases and terminal oxidases (Kranz et al., 2009). Together, the co-occurrence of cbb3-type, Cyt-bd, and Cyt-c enzymes supports the idea that these microbial communities are well-adapted to the fluctuating redox conditions typical of shallow-water hydrothermal vent systems (Borisov et al., 2021).

The predominance of the chemolithoautotrophic community, dominated by microorganisms of the Epsilonproteobacteria class and, more specifically, by the *Sulfurimonas* genus, highlights their key roles in the ecosystem functioning. Our sequence data depict a metabolic framework in which carbon fixation likely occurs alongside nitrate reduction, as already suggested by Tang et al. (2013) and observations made on chemoautotrophy in a deep-sea context (Sievert and Vetriani, 2012). This result is in line with what we observe in other studies performed on Milos shallow water vents (Lu et al., 2020; Sievert et al., 1999; Sievert et al., 2000; Giovannelli et al., 2013). While previous research has highlighted the dominance of Epsilonproteobacteria and their role in sulfur and carbon cycling, our findings further emphasize the functional adaptation of these microbial communities to low-nitrate environments and their genetic potential to oxidize molecular hydrogen (H_2_) as a potential energy source, although hydrogen sulfide (H_2_S) is likely the dominant electron donor in this environment.

We also detected viral genes in the white microbial mat community (Supplementary Figure S2) that could promote the transfer or exchange of genetic material (Jarett et al., 2020; Carreira et al., 2020). The integrase genes identified in our reads facilitate horizontal gene transfer by enabling the integration and excision of genetic elements, thereby promoting genetic diversity and adaptation to environmental stressors (Springael and Top, 2004). The high abundance of CRISPRs and Cas-related genes suggest that microbes in our samples developed a defense system against viral activity (Heidelberg et al., 2009), thus pointing to viral-host interactions and coevolution as a driver of microbial genetic diversity in hydrothermal vents ecosystems (Andersson and Banfield, 2008). Moreover, a high number of transposable element (TE) has been found in our reads, which can be significant in extreme environments, serving as a signal of cell stress and consequently facilitating movement of functional genes between genomes, thus serving as powerful means of adaptation (Vigil-Stenman et al., 2017). Specifically, in extreme environments such as hydrothermal vents, TEs play a crucial role in microbial evolution by facilitating genome plasticity, enabling rapid adaptation to fluctuating conditions, and increasing genetic diversity (Piednoël and Bonnivard, 2009). By mediating horizontal gene transfer, TEs can help microbes acquire beneficial traits, such as heavy metal resistance or enhanced metabolic capabilities, which are essential for survival in these extreme habitats (Casacuberta and González, 2013; Prieto-Barajas et al., 2018). The dense microbial aggregation within the mat creates microhabitats with distinct physicochemical gradients, fostering niche specialization and supporting a high diversity of metabolic functions (Meier et al., 2017). This structural complexity not only promotes microbial diversity but also enhances ecological resilience by enabling the exchange of adaptive genes through horizontal gene transfer, allowing microbes to rapidly respond to environmental fluctuations and stressors (Ghosh et al., 2015). In summary, our study reveals the presence of viral-associated genes and transposable elements, suggesting a potential for dynamic genetic exchange within the mat community, though further analysis is required to assess co-localization with functional genes.

### 4.3 White microbial mats are resilient to storm events

The hydrothermal activity reported in this region is linked to the mixing between the hydrothermal fluids and seawater (Yücel et al., 2013). A previous study on the shallow-water hydrothermal systems of the Gulf of Naples showed how different fluid mixing played a determining role in constraining microbial community structure in shallow-water hydrothermal systems (Bellec et al., 2020). Similarly, the distribution of the white mats of Milos islands is strongly influenced by hydrothermal fluid and seawater mixing patterns (Yücel et al., 2013), and by episodic hydrodynamic events (i.e., storms) which determine a shift in the mixing pattern and the transient disappearance of the white mats (Yücel et al., 2013).

The increase of *Sulfurimonas* related sequences with respect to *Arcobacter* during and after the storm (i.e., after a disturbance event) may reflect its resilience and competitive advantage under the new environmental conditions, possibly driven by temperature shifts, sulfide dynamics, and possibly other electron donor availability (e.g., hydrogen). These factors may have favored *Sulfurimonas* as an early colonizer in the re-establishing community, although the specific drivers of this shift remain difficult to untangle. This interpretation is supported by environmental data from Yücel et al. (2013), which were collected concurrently with the microbial samples analyzed in this study. Three days after the storm event, the microbial white mat was once again thick (up and over 1 cm) and the community diversified further, suggestive of an ecological succession already described in shallow-water vents of Tor Caldara (Patwardhan et al., 2018), and deep-sea hydrothermal vent chemosynthetic biofilm colonization (O’Brien et al., 2015). Species belonging to *Sulfurovum* have been found in sulfidic habitats worldwide, including deep-sea and shallow-water hydrothermal vents (Dahle et al., 2013; Mino et al., 2014; Price and Giovannelli, 2017), and were reported in this study to be the most abundant bacterial genus at the late stage of storm recovery. The microbial community composition 3 days post storm was the most diverse, compared to both the mature community before, and the recently established community right after the storm event. This suggests that the storm (along with the following increased fluid mixing) and the re-establishment of the microbial mat, could have allowed the replenishment of essential nutrients (for instance, nitrogen species and organic carbon), expanding the ecological niches to be explored by the microbial communities, and therefore selecting for a more diverse microbial community in line with prediction from the intermediate disturbance theory (Giguère and Tunnicliffe, 2021). These temporarily available niches become exhausted overtime, and *Arcobacter* spp. become dominant prior to the next disturbance cycle. Disturbance events, such as storms and tides, with their transient nature, exert influence on the environment and, as a consequence, on the resident microbial communities. Such results underscore the interplay among episodic hydrodynamic events, geochemical gradients and niche differentiation created by the mixing of seawater and vent fluids and by disturbance events. These factors emerge as critical determinants shaping the dynamics of benthic microbial communities in shallow-water hydrothermal vents and additional research with focused time-series studies is necessary for an in-depth understanding of these processes.

### 4.4 Benthic boundary layer interactions shape the spatial distribution of microbial mats in shallow vents

Sand ripples in shallow-water hydrothermal vent fields such as Paleochori Bay are formed by the interaction between bottom currents and unconsolidated sediment. These bedforms arise as flow instabilities develop when current velocity exceeds a threshold, causing sediment to be entrained, transported, and redeposited in a rhythmic pattern (Blondeaux, 1990). In hydrothermal settings, these ripples are not only shaped by hydrodynamics due to current and waves, but also by hydrothermal fluid advection and the complex interaction between the two influence fluid–sediment interactions, creating microtopographies that affect both geochemical gradients and microbial community structure.

The sampled Milos microbial mats were characterized by a stratified layering of mat colors within a sand ripple near the venting location. Mat coloration transitioned from yellow at the base, through Siena red in the middle, to brown moving from the trough to the crest and were present only on one side of the ripple (Figure 7B; Supplementary Figure S3). These three colored mat layers overlaid a foundational white mat. The composition of the community varied according to the color, with the relative abundance of Cyanobacteria increasing along the side of the ripple and having the highest abundances in the brown mats at the top of the ripple (Figure 7B). A possible explanation for this distribution is the exposure to sunlight (facing south) on one of the ripple sides favoring phototrophic microorganisms. This hypothesis can be quickly eliminated as the ripples changed in direction during our sampling campaign in Milos, and the coloring of the mats was always present on the leading edge of the ripple, with respect to the main currents generating the ripples, rather than facing the same bearing. We therefore hypothesized that complex fluid interactions between the buoyant hydrothermal fluids, the dominant currents and the porous sandy bottom might account for the differential microbial distribution observed. In porous sediments the diffusion of O_2_ can be modulated by the interactions of the benthic boundary layer with the sandy bottom (Jørgensen, 1994). The flow of seawater over the sandy sediments in the presence of sand ripple can favour the advective flow of deeper pore water (Precht and Huettel, 2004). This would contribute to a velocity gradient of oxygenated water through the sandy structure, which is reported to be lower at the basis of the ripple and higher in correspondence of the crest (Precht and Huettel, 2004). In addition, the presence of microbial mats, together with the porosity of the sediments, could affect the flow through sandy structures (Jørgensen and Revsbech, 1985; Precht and Huettel, 2004).

The Milos system is shaped by the interplay between lateral seawater flow, driven by tides and wave action, and the buoyant ascent of hydrothermal fluids characteristic of shallow-water hydrothermal vents. This interaction creates complex sediment structures such as sand ripples, which in turn generate dynamic boundary layer conditions influencing both fluid mixing and microbial colonization. In our initial modeling experiments, using a computational method known as the lattice Boltzmann approach, we explored how water flow interacts with a simplified sediment ripple shape (Supplementary Figure S1). We found that when water moves over this ripple structure, it creates a swirling, recirculating flow pattern—known as an eddy—just behind the ripple. This phenomenon occurs due to a process called boundary layer separation. Boundary layer separation happens when flowing water near a solid surface, such as the ripple, slows down and detaches from the surface, forming turbulent vortices or eddies downstream.

These eddies are important because they significantly influence how substances dissolved in water (known as passive scalars, such as nutrients or chemical signals) mix and distribute in the area around the ripple. Additionally, they control the transport and deposition of small particles, which could include sediment grains or microorganisms.

Interestingly, the microbial communities present in the sediment may also interact with these flow patterns. Microbes might alter the local environment, potentially changing sediment stability or flow characteristics, while at the same time, the flow could affect where and how microbes grow and thrive.

In future studies, we plan to expand our modeling to capture more complex interactions. This will include tracking how particles move within these flow patterns, understanding how erosion and deposition processes reshape the ripple structures, and examining how microbial communities directly impact the physical structure of the sediment and thereby influence the overall flow dynamics. When using combined FVM and LBM fluid flow to reconstruct the possible interactions in the Milos microbial mats system, our results show that, at the small scale of a single sand ripple, the primary mechanism driving the temperature-redox gradient is the mixing of hydrothermal fluids with the overlying seawater. This process, which governs the availability of electron donors and acceptors for the *in situ* microbial community, is controlled by the relative speed of the lateral seawater flow. In conditions where lateral flow is reduced or near zero, the advection of hydrothermal fluids dominate the system creating complex microcirculation cells near the vent center (Yücel et al., 2013). This pressure-driven phenomenon, observed previously by Yücel et al. (2013) using in situ microsensors, generates an inward flux of oxygenated seawater in the area surrounding the vent center. When lateral flow of seawater reaches a threshold velocity capable of overcoming and interacting with the advective flow, complex boundary layer dynamics control mixing (Supplementary Figure S1). We observed the presence of a positive pressure area in the stoss side of the sand ripple, favouring the oxygenated, cold seawater flux in the ripple side and crest. This might lead to higher re-circulation of nutrients and oxygenated water, lower temperatures and lower concentrations of sulfide favouring the presence of microorganisms involved in aerobic respiration and photosynthesis (Figure 7A). Our results support this conclusion as the relative abundance of *Cyanobacteria* increases over the ripple stoss side, reaching its peak at the ripples crest, while the abundance of chemolithotrophic sulfur oxidizers like *Sulfurimonas* and related Epsilonproteobacteria genera decreases.

On the lee side of the sand ripple, the negative pressure generated by the lateral flow of seawater induces an increased flux of hydrothermal fluids, generating a pocket of higher temperature, reduced fluids in the trough between ripples. Microbial diversity data shows at this location the community is dominated by *Sulfurimonas* and related Epsilonproteobacteria genera, consistent with their physiology and preferred metabolisms (Campbell et al., 2006). The complex dynamics between microbial distribution and fluid dynamic interactions over the seafloor has been overlooked as a possible explanatory variable in describing small scale variability, especially in shallow-water hydrothermal vents (Esposito et al., 2018). Overall our results highlight the importance of considering the effects of dynamic events, both episodic events like storms and complex dynamic mixing across small scales to comprehend the factors controlling microbial community structuring in natural systems.

## 5 Conclusion

Our study demonstrates that hydrodynamic forces play a fundamental role in shaping microbial communities in the white mats of Milos shallow-water hydrothermal vents. These mat communities are dominated by chemolithoautotrophic *Epsilonproteobacteria* that thrive on the chemical energy of vent fluids, yet their population structure is highly dynamic in space and time. We found that a major storm disturbance caused a rapid but transient reorganization of the community, followed by a succession that restored the mat’s characteristic sulfur-oxidizer dominance within days. Spatially, small-scale variations in fluid mixing created a patchwork of niches across the vent field: for example, oxygen-rich microhabitats generated by benthic boundary layer flow dynamics supported cyanobacterial growth in the sand ripple crests, whereas sulfide-rich small scale zones selected for *Sulfurimonas* and *Arcobacter*. Together, these results highlight that both transient events acting on a large spatial scale (storms) and persistent physical gradients affecting the local niches (flow and diffusion across the benthic boundary layer) drive the diversity and function of vent-associated biofilms. The close coupling between hydrodynamic processes (fluid flow, tides, waves and storms) and microbial ecology controls the distribution and contribution of these ecosystems to biogeochemical cycling, both at the local and regional scale. Understanding this interplay is crucial for predicting how shallow vent ecosystems respond to natural disturbances and environmental changes. Our findings provide a framework for how geodynamic events influence microbial succession and resilience in coastal hydrothermal systems, and underscore the need for further studies of these dynamic habitats. Future work targeting shallow-water vents will help refine our models of biogeochemical feedback in these systems and shed additional light on the strategies microbial communities use to persist amidst environmental variability.

## Data Availability

The original contributions presented in the study are publicly available. This data can be found here: https://www.ebi.ac.uk/ena/browser/home, accession number PRJEB8854. All data and code used in this paper is available on the GitHub repository: https://github.com/giovannellilab/Milos_white_mats and made available through Zenodo at: 10.5281/zenodo.15528839.

## Footnotes

^1^ http://www.mrdnalab.com

## Supplementary material

SUPPLEMENTARY FIGURE S1 **(A)** Streamwise velocity component field deriving from FVM model, [m/s]. Flow direction starting from left to right of the modelling mesh. The modeling domain is constrained within a 45x55 cm box. **(B)** Transverse velocity component field deriving from FVM model, [m/s]. **(C)** Steady state flow structure of our modelled ripple and porous bed. The units here are simply the number of simulation grid points, so the numerical simulation grid in this case was 5000x10000 grid points in size. These units are dimensionless as the only relevant parameters in the model are dimensionless number groups such as the Reynolds number (a dimensionless quantity in fluid dynamics that helps predict the flow pattern of a fluid), Rayleigh number (a dimensionless quantity that characterizes the buoyancy-driven flow, or free convection, of a fluid), Nusselt number (dimensionless number that describes the ratio of convective heat transfer to conductive heat transfer at the boundary between a solid surface and a fluid). Upper panel: fluid density, second panel: horizontal flow rate, third panel: vertical flow rate, lower panel: temperature. Steady fluid motion is imposed using a constant horizontal shear flow at the upper boundary, as seen in the colormap of horizontal momentum [second panel of **(C)**]. In the third panel we see the vertical velocity component, w. The lower panel shows temperature: convection has little impact in this particular configuration, but thermal energy is being advected by the flow, namely the boundary layer separation effect downstream of the ripple.

SUPPLEMENTARY FIGURE S2 Viral diversity in the microbial mat metagenome classified according to the Baltimore classification (inner circle), and the International Commission on Viral Taxonomy classification at the Order (median circle) and Family (outer circle) level. A total of 30,891 sequences were classified using MetaVIR with an e-value cutoff of 10^−5^ (a total of 50,050 sequences were classified using a less conservative e-value cutoff of 10^−3^).

SUPPLEMENTARY FIGURE S3 Photograph of microbial mats at the hydrothermal venting area off Milos Island, Greece. The image shows a series of sand ripples coated with microbial mats displaying distinct surface coloration gradients: white, yellow, light reddish/orangish and brownish, with yellow mats often overlying white mats along ripple flanks. These mat colors reflect differences in microbial community composition and are consistent with stratified layering represented in Figure 7B. The coloration and distribution are influenced by venting intensity and hydrodynamic flow conditions. Image credit: Thanos Dailianis/Hellenic Centre for Marine Research (SeaBioTech project, FP7-KBBE.2012.3.2-01).

## References

[ref1] Alain K. Zbinden M. Le Bris N. Lesongeur F. Quérellou J. Gaill F.. (2004). Early steps in microbial colonization processes at deep-sea hydrothermal vents. Environ. Microbiol. 6, 227–241. doi: 10.1111/j.1462-2920.2003.00557.x, PMID: 14871207

[ref2] Andersson A. F. Banfield J. F. (2008). Virus population dynamics and acquired virus resistance in natural microbial communities. Science 320, 1047–1050. doi: 10.1126/science.1157358, PMID: 18497291

[ref3] Arcadi E. Rizzo C. Calogero R. Sciutteri V. Fabiano F. Consoli P.. (2023). Microbial communities inhabiting shallow hydrothermal vents as sentinels of acidification processes. Front. Microbiol. 14:1233893. doi: 10.3389/fmicb.2023.1233893, PMID: 37727286 PMC10505797

[ref4] Bellec L. Cambon-Bonavita M.-A. Durand L. Aube J. Gayet N. Sandulli R.. (2020). Microbial communities of the shallow-water hydrothermal vent near Naples, Italy, and chemosynthetic symbionts associated with a free-living marine nematode. Front. Microbiol. 11:2023. doi: 10.3389/fmicb.2020.02023, PMID: 32973733 PMC7469538

[ref5] Bengtsson J. Eriksson K. M. Hartmann M. Wang Z. Shenoy B. D. Grelet G.-A.. (2011). Metaxa: a software tool for automated detection and discrimination among ribosomal small subunit (12S/16S/18S) sequences of archaea, bacteria, eukaryotes, mitochondria, and chloroplasts in metagenomes and environmental sequencing datasets. Antonie Van Leeuwenhoek 100, 471–475. doi: 10.1007/s10482-011-9598-6, PMID: 21674231

[ref6] Blondeaux P. (1990). Sand ripples under sea waves part 1. Ripple formation. J. Fluid Mech. 218, 1–17. doi: 10.1017/S0022112090000908

[ref7] Borisov V. B. Gennis R. B. Hemp J. Verkhovsky M. I. (2011). The cytochrome *bd* respiratory oxygen reductases. Biochimica et Biophysica Acta (BBA) 1807, 1398–1413. doi: 10.1016/j.bbabio.2011.06.016, PMID: 21756872 PMC3171616

[ref8] Borisov V. B. Siletsky S. A. Paiardini A. Hoogewijs D. Forte E. Giuffrè A.. (2021). Bacterial oxidases of the cytochrome bd family: redox enzymes of unique structure, function, and utility as drug targets. Antioxid. Redox Signal. 34, 1280–1318. doi: 10.1089/ars.2020.8039, PMID: 32924537 PMC8112716

[ref9] Botz R. Stüben D. Winckler G. Bayer R. Schmitt M. Faber E. (1996). Hydrothermal gases offshore Milos Island, Greece. Chem. Geol. 130, 161–173. doi: 10.1016/0009-2541(96)00023-X

[ref10] Campbell B. J. Engel A. S. Porter M. L. Takai K. (2006). The versatile ε-proteobacteria: key players in sulphidic habitats. Nat. Rev. Microbiol. 4, 458–468. doi: 10.1038/nrmicro1414, PMID: 16652138

[ref11] Caporaso J. G. Kuczynski J. Stombaugh J. Bittinger K. Bushman F. D. Costello E. K.. (2010). Qiime allows analysis of high-throughput community sequencing data. Nat. Methods 7, 335–336. doi: 10.1038/nmeth.f.303, PMID: 20383131 PMC3156573

[ref12] Cardigos F. Colaço A. Dando P. R. Ávila S. P. Sarradin P.-M. Tempera F.. (2005). Shallow water hydrothermal vent field fluids and communities of the D. João de Castro seamount (Azores). Chem. Geol. 224, 153–168. doi: 10.1016/j.chemgeo.2005.07.019

[ref13] Carreira C. Lønborg C. Kühl M. Lillebø A. I. Sandaa R.-A. Villanueva L.. (2020). Fungi and viruses as important players in microbial mats. FEMS Microbiol. Ecol. 96:fiaa187. doi: 10.1093/femsec/fiaa187, PMID: 32966583

[ref14] Casacuberta E. González J. (2013). The impact of transposable elements in environmental adaptation. Mol. Ecol. 22, 1503–1517. doi: 10.1111/mec.12170, PMID: 23293987

[ref15] Chen M. Li Y. Tang K. Hu A. Fan W. Wang D.. (2023). Highly diverse diazotrophs drive high N2 fixation rates in a shallow submarine hydrothermal system. Fundam. Res. doi: 10.1016/j.fmre.2023.07.009 PMC13247510 42272469

[ref16] Chen X. Tang K. Zhang M. Liu S. Chen M. Zhan P.. (2022). Genome-centric insight into metabolically active microbial population in shallow-sea hydrothermal vents. Microbiome 10:170. doi: 10.1186/s40168-022-01351-7, PMID: 36242065 PMC9563475

[ref17] Chun J. Lee J.-H. Jung Y. Kim M. Kim S. Kim B. K.. (2007). Eztaxon: a web-based tool for the identification of prokaryotes based on 16S ribosomal RNA gene sequences. Int. J. Syst. Evol. Microbiol. 57, 2259–2261. doi: 10.1099/ijs.0.64915-0, PMID: 17911292

[ref18] Dahl C. (2017). “Sulfur metabolism in phototrophic Bacteria” in Modern topics in the phototrophic prokaryotes: Metabolism, bioenergetics, and omics. ed. Hallenbeck P. C. (Cham: Springer International Publishing), 27–66.

[ref19] Dahle H. Roalkvam I. Thorseth I. H. Pedersen R. B. Steen I. H. (2013). The versatile in situ gene expression of an Epsilonproteobacteria-dominated biofilm from a hydrothermal chimney. Environ. Microbiol. Rep. 5, 282–290. doi: 10.1111/1758-2229.12016, PMID: 23584970

[ref20] Dando P. R. (2010). “Biological communities at marine shallow-water vent and seep sites” in The vent and seep biota: Aspects from microbes to ecosystems. ed. Kiel S. (Dordrecht: Springer Netherlands), 333–378.

[ref21] Dando P. R. Aliani S. Arab H. Bianchi C. N. Brehmer M. Cocito S.. (2000). Hydrothermal studies in the Aegean Sea. Phys. Chem. Earth 25, 1–8. doi: 10.1016/S1464-1909(99)00112-4

[ref22] Dando P. R. Hughes J. A. Leahy Y. Niven S. J. Taylor L. J. Smith C. (1995). Gas venting rates from submarine hydrothermal areas around the island of Milos, Hellenic volcanic arc. Cont. Shelf Res. 15, 913–929. doi: 10.1016/0278-4343(95)80002-U

[ref23] Dando P. R. Stüben D. Varnavas S. P. (1999). Hydrothermalism in the Mediterranean Sea. Prog. Oceanogr. 44, 333–367. doi: 10.1016/S0079-6611(99)00032-4

[ref24] Dobretsov S. (2009). “Marine biofilms” in Biofouling (John Wiley & Sons, Ltd.), 123–136.

[ref25] Douglas S. (2000). Environmental scanning electron microscopy studies of colloidal sulfur deposition in a natural microbial community from a cold sulfide spring near Ancaster, Ontario, Canada. Geomicrobiol J. 17, 275–289. doi: 10.1080/01490450050192974

[ref26] Dowd S. E. Sun Y. Wolcott R. D. Domingo A. Carroll J. A. (2008). Bacterial tag–encoded FLX amplicon pyrosequencing (bTEFAP) for microbiome studies: bacterial diversity in the ileum of newly weaned Salmonella-infected pigs. Foodborne Pathog. Dis. 5, 459–472. doi: 10.1089/fpd.2008.0107, PMID: 18713063

[ref27] Edgar R. C. (2010). Search and clustering orders of magnitude faster than BLAST. Bioinformatics 26, 2460–2461. doi: 10.1093/bioinformatics/btq461, PMID: 20709691

[ref28] Edgar R. C. Haas B. J. Clemente J. C. Quince C. Knight R. (2011). Uchime improves sensitivity and speed of chimera detection. Bioinformatics 27, 2194–2200. doi: 10.1093/bioinformatics/btr381, PMID: 21700674 PMC3150044

[ref29] Edwards R. Olson R. Disz T. (n.d.). Real time metagenomics: using k-mers to annotate metagenomes | bioinformatics | Oxford academic. Available online at: https://academic.oup.com/bioinformatics/article/28/24/3316/245032 (Accessed February 2, 2023). 10.1093/bioinformatics/bts599 PMC3519453 23047562

[ref30] Esposito V. Andaloro F. Canese S. Bortoluzzi G. Bo M. Bella M. D.. (2018). Exceptional discovery of a shallow-water hydrothermal site in the SW area of Basiluzzo islet (Aeolian archipelago, South Tyrrhenian Sea): an environment to preserve. PLoS One 13:e0190710. doi: 10.1371/journal.pone.0190710, PMID: 29300784 PMC5754086

[ref31] Fitzsimons M. f. Dando P. R. Hughes J. A. Thiermann F. Akoumianaki I. Pratt S. M. (1997). Submarine hydrothermal brine seeps off Milos, Greece. Observations and geochemistry. Mar. Chem. 57, 325–340. doi: 10.1016/S0304-4203(97)00021-2

[ref32] Flemming H.-C. Wingender J. (2010). The biofilm matrix. Nat. Rev. Microbiol. 8, 623–633. doi: 10.1038/nrmicro2415, PMID: 20676145

[ref33] Frawley E. R. Fang F. C. (2014). The ins and outs of bacterial iron metabolism. Mol. Microbiol. 93, 609–616. doi: 10.1111/mmi.12709, PMID: 25040830 PMC4135372

[ref34] Friedrich C. G. Bardischewsky F. Rother D. Quentmeier A. Fischer J. (2005). Prokaryotic sulfur oxidation. Curr. Opin. Microbiol. 8, 253–259. doi: 10.1016/j.mib.2005.04.005, PMID: 15939347

[ref35] Fuchs G. (2011). Alternative pathways of carbon dioxide fixation: insights into the early evolution of life? Ann. Rev. Microbiol. 65, 631–658. doi: 10.1146/annurev-micro-090110-102801, PMID: 21740227

[ref36] Garnett J. A. Matthews S. (2012). Interactions in bacterial biofilm development: a structural perspective. Curr. Protein Pept. Sci. 13, 739–755. doi: 10.2174/138920312804871166, PMID: 23305361 PMC3601411

[ref37] Ghosh W. Mallick S. DasGupta S. K. (2009). Origin of the sox multienzyme complex system in ancient thermophilic bacteria and coevolution of its constituent proteins. Res. Microbiol. 160, 409–420. doi: 10.1016/j.resmic.2009.07.003, PMID: 19616092

[ref38] Ghosh W. Roy C. Roy R. Nilawe P. Mukherjee A. Haldar P. K.. (2015). Resilience and receptivity worked in tandem to sustain a geothermal mat community amidst erratic environmental conditions. Sci. Rep. 5:12179. doi: 10.1038/srep12179, PMID: 26184838 PMC4505329

[ref39] Giguère T. N. Tunnicliffe V. (2021). Beta diversity differs among hydrothermal vent systems: implications for conservation. PLoS One 16:e0256637. doi: 10.1371/journal.pone.0256637, PMID: 34437606 PMC8389485

[ref40] Giovannelli D. d’Errico G. Manini E. Yakimov M. Vetriani C. (2013). Diversity and phylogenetic analyses of bacteria from a shallow-water hydrothermal vent in Milos island (Greece). Front. Microbiol. 4:184. doi: 10.3389/fmicb.2013.00184 23847607 PMC3703532

[ref41] Giovannelli D. Grosche A. Starovoytov V. Yakimov M. Manini E. Vetriani C. (2012). *Galenea microaerophila* gen. Nov., sp. nov., a mesophilic, microaerophilic, chemosynthetic, thiosulfate-oxidizing bacterium isolated from a shallow-water hydrothermal vent. Int. J. Syst. Evol. Microbiol. 62, 3060–3066. doi: 10.1099/ijs.0.040808-0, PMID: 22307509

[ref88] Giovannelli D. Price R. E. (2019). “Marine shallow-water hydrothermal vents: geochemistry” in Encyclopedia of Ocean Sciences (Third Edition). eds. Cochran J. K. Bokuniewicz H. J. Yager P. L. (Oxford: Academic Press), 353–363.

[ref42] Gledhill M. Buck K. N. (2012). The organic complexation of iron in the marine environment: a review. Front. Microbiol. 3:69. doi: 10.3389/fmicb.2012.00069, PMID: 22403574 PMC3289268

[ref43] Gomez-Saez G. V. Pop Ristova P. Sievert S. M. Elvert M. Hinrichs K.-U. Bühring S. I. (2017). Relative importance of chemoautotrophy for primary production in a light exposed marine shallow hydrothermal system. Front. Microbiol. 8:702. doi: 10.3389/fmicb.2017.00702, PMID: 28484442 PMC5399606

[ref44] Greenshields C. J. Weller H. G. (2022). Notes on computational fluid dynamics: general principles. CFD Direct. Available online at: https://cir.nii.ac.jp/crid/1130578038682879362 (Accessed May 25, 2025).

[ref45] Grote J. Schott T. Bruckner C. G. Glöckner F. O. Jost G. Teeling H.. (2012). Genome and physiology of a model *Epsilonproteobacterium* responsible for sulfide detoxification in marine oxygen depletion zones. Proc. Natl. Acad. Sci. USA 109, 506–510. doi: 10.1073/pnas.1111262109, PMID: 22203982 PMC3258601

[ref46] Groth A. C. Calos M. P. (2004). Phage integrases: biology and applications. J. Mol. Biol. 335, 667–678. doi: 10.1016/j.jmb.2003.09.082, PMID: 14687564

[ref47] Gulmann L. K. Beaulieu S. E. Shank T. M. Ding K. Seyfried W. E. Sievert S. M. (2015). Bacterial diversity and successional patterns during biofilm formation on freshly exposed basalt surfaces at diffuse-flow deep-sea vents. Front. Microbiol. 6:901. doi: 10.3389/fmicb.2015.00901, PMID: 26441852 PMC4564720

[ref48] Hall-Stoodley L. Costerton J. W. Stoodley P. (2004). Bacterial biofilms: from the natural environment to infectious diseases. Nat. Rev. Microbiol. 2, 95–108. doi: 10.1038/nrmicro821, PMID: 15040259

[ref49] Han Y. Perner M. (2015). The globally widespread genus *Sulfurimonas*: versatile energy metabolisms and adaptations to redox clines. Front. Microbiol. 6:989. doi: 10.3389/fmicb.2015.00989, PMID: 26441918 PMC4584964

[ref50] Heidelberg J. F. Nelson W. C. Schoenfeld T. Bhaya D. (2009). Germ warfare in a microbial mat community: CRISPRs provide insights into the co-evolution of host and viral genomes. PLoS One 4:e4169. doi: 10.1371/journal.pone.0004169, PMID: 19132092 PMC2612747

[ref51] Holmström C. Kjelleberg S. (1999). Marine *Pseudoalteromonas* species are associated with higher organisms and produce biologically active extracellular agents. FEMS Microbiol. Ecol. 30, 285–293. doi: 10.1111/j.1574-6941.1999.tb00656.x 10568837

[ref52] Howard-Varona C. Lindback M. M. Bastien G. E. Solonenko N. Zayed A. A. Jang H.. (2020). Phage-specific metabolic reprogramming of virocells. ISME J. 14, 881–895. doi: 10.1038/s41396-019-0580-z, PMID: 31896786 PMC7082346

[ref53] Huang F. Pan L. He Z. Zhang M. Zhang M. (2020). Identification, interactions, nitrogen removal pathways and performances of culturable heterotrophic nitrification-aerobic denitrification bacteria from mariculture water by using cell culture and metagenomics. Sci. Total Environ. 732:139268. doi: 10.1016/j.scitotenv.2020.139268, PMID: 32402929

[ref54] Huber J. A. Butterfield D. A. Baross J. A. (2003). Bacterial diversity in a subseafloor habitat following a deep-sea volcanic eruption. FEMS Microbiol. Ecol. 43, 393–409. doi: 10.1111/j.1574-6941.2003.tb01080.x, PMID: 19719671

[ref55] Hügler M. Sievert S. M. (2011). Beyond the Calvin cycle: autotrophic carbon fixation in the ocean. Annu. Rev. Mar. Sci. 3, 261–289. doi: 10.1146/annurev-marine-120709-142712, PMID: 21329206

[ref56] Hügler M. Wirsen C. O. Fuchs G. Taylor C. D. Sievert S. M. (2005). Evidence for autotrophic CO2 fixation via the reductive tricarboxylic acid cycle by members of the ɛ subdivision of Proteobacteria. J. Bacteriol. 187, 3020–3027. doi: 10.1128/JB.187.9.3020-3027.2005, PMID: 15838028 PMC1082812

[ref57] Hyatt D. Chen G.-L. LoCascio P. F. Land M. L. Larimer F. W. Hauser L. J. (2010). Prodigal: prokaryotic gene recognition and translation initiation site identification. BMC Bioinform. 11:119. doi: 10.1186/1471-2105-11-119, PMID: 20211023 PMC2848648

[ref58] Jarett J. K. Džunková M. Schulz F. Roux S. Paez-Espino D. Eloe-Fadrosh E.. (2020). Insights into the dynamics between viruses and their hosts in a hot spring microbial mat. ISME J. 14, 2527–2541. doi: 10.1038/s41396-020-0705-4, PMID: 32661357 PMC7490370

[ref59] Jørgensen B. B. (1994). Sulfate reduction and thiosulfate transformations in a cyanobacterial mat during a diel oxygen cycle. FEMS Microbiol. Ecol. 13, 303–312. doi: 10.1111/j.1574-6941.1994.tb00077.x

[ref60] Jørgensen B. B. Revsbech N. P. (1985). Diffusive boundary layers and the oxygen uptake of sediments and detritus. Limnol. Oceanogr. 30, 111–122. doi: 10.4319/lo.1985.30.1.0111

[ref61] Kamenev G. M. Fadeev V. I. Selin N. I. Tarasov V. G. Malakhov V. V. (1993). Composition and distribution of macro- and meiobenthos around sublittoral hydrothermal vents in the Bay of Plenty, New Zealand. N. Z. J. Mar. Freshw. Res. 27, 407–418. doi: 10.1080/00288330.1993.9516582

[ref62] Kanehisa M. Goto S. (2000). KEGG: Kyoto encyclopedia of genes and genomes. Nucleic Acids Res. 28, 27–30. doi: 10.1093/nar/28.1.27 10592173 PMC102409

[ref63] Khimasia A. Rovere A. Pichler T. (2020). Hydrothermal areas, microbial mats and sea grass in Paleochori Bay, Milos, Greece. J. Maps 16, 348–356. doi: 10.1080/17445647.2020.1748131

[ref9001] Kokkalas S. Xypolias P. Koukouvelas I. Doutsos T. (2006). Post-collisional contractional and extensional deformation in the Aegean region. In: Post-Collisional Tectonics and Magmatism in the Mediterranean Region and Asia. Geological Society of America Special Paper. (eds). Dilek Y. Pavlides S.. 409, pp 97–123.

[ref64] Kranz R. G. Richard-Fogal C. Taylor J.-S. Frawley E. R. (2009). Cytochrome c biogenesis: mechanisms for covalent modifications and trafficking of heme and for heme-iron redox control. Microbiol. Mol. Biol. Rev. 73, 510–528. doi: 10.1128/mmbr.00001-09 19721088 PMC2738134

[ref65] Kümpel C. Grein F. Dahl C. (2023). Fluorescence microscopy study of the intracellular sulfur globule protein SgpD in the purple sulfur bacterium *Allochromatium vinosum*. Microorganisms 11:1792. doi: 10.3390/microorganisms11071792, PMID: 37512964 PMC10386293

[ref66] Labrenz M. Grote J. Mammitzsch K. Boschker H. T. S. Laue M. Jost G.. (2013). *Sulfurimonas* gotlandica sp. nov., a chemoautotrophic and psychrotolerant epsilonproteobacterium isolated from a pelagic redoxcline, and an emended description of the genus Sulfurimonas. Int. J. Syst. Evol. Microbiol. 63, 4141–4148. doi: 10.1099/ijs.0.048827-0, PMID: 23749282 PMC3836495

[ref67] Lagesen K. Hallin P. Rødland E. A. Staerfeldt H.-H. Rognes T. Ussery D. W. (2007). RNAmmer: consistent and rapid annotation of ribosomal RNA genes. Nucleic Acids Res. 35, 3100–3108. doi: 10.1093/nar/gkm160, PMID: 17452365 PMC1888812

[ref68] Le Moine Bauer S. Lu G.-S. Goulaouic S. Puzenat V. Schouw A. Barreyre T.. (2023). Structure and metabolic potential of the prokaryotic communities from the hydrothermal system of Paleochori Bay, Milos, Greece. Front. Microbiol. 13:1060168. doi: 10.3389/fmicb.2022.1060168, PMID: 36687571 PMC9852839

[ref69] Lentini V. Gugliandolo C. Bunk B. Overmann J. Maugeri T. L. (2014). Diversity of prokaryotic community at a shallow marine hydrothermal site elucidated by Illumina sequencing technology. Curr. Microbiol. 69, 457–466. doi: 10.1007/s00284-014-0609-5, PMID: 24849732

[ref70] Lu G.-S. LaRowe D. E. Fike D. A. Druschel G. K. Iii W. P. G. Price R. E.. (2020). Bioenergetic characterization of a shallow-sea hydrothermal vent system: Milos Island, Greece. PLoS One 15:e0234175. doi: 10.1371/journal.pone.0234175 32502166 PMC7274409

[ref71] McGinnis S. Madden T. L. (2004). BLAST: at the core of a powerful and diverse set of sequence analysis tools. Nucleic Acids Res. 32, W20–W25. doi: 10.1093/nar/gkh435, PMID: 15215342 PMC441573

[ref72] Mehta M. P. Butterfield D. A. Baross J. A. (2003). Phylogenetic diversity of nitrogenase (nifH) genes in deep-sea and hydrothermal vent environments of the Juan de Fuca ridge. Appl. Environ. Microbiol. 69, 960–970. doi: 10.1128/AEM.69.2.960-970.2003, PMID: 12571018 PMC143675

[ref73] Meier D. V. Pjevac P. Bach W. Hourdez S. Girguis P. R. Vidoudez C.. (2017). Niche partitioning of diverse sulfur-oxidizing bacteria at hydrothermal vents. ISME J. 11, 1545–1558. doi: 10.1038/ismej.2017.37, PMID: 28375213 PMC5520155

[ref74] Meyer F. Paarmann D. D'Souza M. Olson R. Glass E. Kubal M.. (2008). The metagenomics RAST server – a public resource for the automatic phylogenetic and functional analysis of metagenomes. BMC Bioinform. 9:386. doi: 10.1186/1471-2105-9-386 PMC2563014 18803844

[ref75] Mino S. Kudo H. Arai T. Sawabe T. Takai K. Nakagawa S. (2014). *Sulfurovum aggregans* sp. nov., a hydrogen-oxidizing, thiosulfate-reducing chemolithoautotroph within the Epsilonproteobacteria isolated from a deep-sea hydrothermal vent chimney, and an emended description of the genus Sulfurovum. Int. J. Syst. Evol. Microbiol. 64, 3195–3201. doi: 10.1099/ijs.0.065094-0, PMID: 24966202

[ref76] Müller A. L. Kjeldsen K. U. Rattei T. Pester M. Loy A. (2015). Phylogenetic and environmental diversity of DsrAB-type dissimilatory (bi)sulfite reductases. ISME J. 9, 1152–1165. doi: 10.1038/ismej.2014.208, PMID: 25343514 PMC4351914

[ref77] Muyzer G. de Waal E. C. Uitterlinden A. G. (1993). Profiling of complex microbial populations by denaturing gradient gel electrophoresis analysis of polymerase chain reaction-amplified genes coding for 16S rRNA. Appl. Environ. Microbiol. 59, 695–700. doi: 10.1128/aem.59.3.695-700.1993, PMID: 7683183 PMC202176

[ref78] Nomikou P. Krassakis P. Kazana S. Papanikolaou D. Koukouzas N. (2021). The volcanic relief within the Kos-Nisyros-Tilos tectonic graben at the eastern edge of the Aegean volcanic arc, Greece and geohazard implications. Geosciences 11:231. doi: 10.3390/geosciences11060231

[ref79] O’Brien C. E. Giovannelli D. Govenar B. Luther G. W. Lutz R. A. Shank T. M.. (2015). Microbial biofilms associated with fluid chemistry and megafaunal colonization at post-eruptive deep-sea hydrothermal vents. Deep Sea Res. Part II Top. Stud. Oceanogr. 121, 31–40. doi: 10.1016/j.dsr2.2015.07.020

[ref80] Patwardhan S. Foustoukos D. I. Giovannelli D. Yücel M. Vetriani C. (2018). Ecological succession of sulfur-oxidizing epsilon- and gammaproteobacteria during colonization of a shallow-water gas vent. Front. Microbiol. 9:2970. doi: 10.3389/fmicb.2018.02970, PMID: 30574130 PMC6291522

[ref81] Patwardhan S. Phan J. Smedile F. Vetriani C. (2023). The genome of *Varunaivibrio sulfuroxidans* strain TC8T, a metabolically versatile alphaproteobacterium from the Tor Caldara gas vents in the Tyrrhenian Sea. Microorganisms 11:1366. doi: 10.3390/microorganisms11061366, PMID: 37374867 PMC10305379

[ref82] Patwardhan S. Smedile F. Giovannelli D. Vetriani C. (2021). Metaproteogenomic profiling of chemosynthetic microbial biofilms reveals metabolic flexibility during colonization of a shallow-water gas vent. Front. Microbiol. 12:638300. doi: 10.3389/fmicb.2021.638300, PMID: 33889140 PMC8056087

[ref83] Pereira I. A. C. Ramos A. R. Grein F. Marques M. C. Da Silva S. M. Venceslau S. S. (2011). A comparative genomic analysis of energy metabolism in sulfate reducing bacteria and archaea. Front. Microbiol. 2:69. doi: 10.3389/fmicb.2011.00069 21747791 PMC3119410

[ref84] Pérez-Rodríguez I. Bohnert K. A. Cuebas M. Keddis R. Vetriani C. (2013). Detection and phylogenetic analysis of the membrane-bound nitrate reductase (Nar) in pure cultures and microbial communities from deep-sea hydrothermal vents. FEMS Microbiol. Ecol. 86, 256–267. doi: 10.1111/1574-6941.12158, PMID: 23889124

[ref85] Piednoël M. Bonnivard E. (2009). DIRS1-like retrotransposons are widely distributed among Decapoda and are particularly present in hydrothermal vent organisms. BMC Evol. Biol. 9:86. doi: 10.1186/1471-2148-9-86, PMID: 19400949 PMC2685390

[ref86] Precht E. Huettel M. (2004). Rapid wave-driven advective pore water exchange in a permeable coastal sediment. J. Sea Res. 51, 93–107. doi: 10.1016/j.seares.2003.07.003

[ref87] Price R. E. Giovannelli D. (2017). “A review of the geochemistry and microbiology of marine shallow-water hydrothermal vents” in Reference module in earth systems and environmental sciences. eds. Cochran J. K. Bokuniewicz H. J. Yager P. L. (Oxford, U.K.: Elsevier). doi: 10.1016/B978-0-12-409548-9.09523-3

[ref89] Price R. E. Lesniewski R. Nitzsche K. S. Meyerdierks A. Saltikov C. Pichler T.. (2013). Archaeal and bacterial diversity in an arsenic-rich shallow-sea hydrothermal system undergoing phase separation. Front. Microbiol. 4:158. doi: 10.3389/fmicb.2013.00158, PMID: 23847597 PMC3705188

[ref90] Prieto-Barajas C. M. Valencia-Cantero E. Santoyo G. (2018). Microbial mat ecosystems: structure types, functional diversity, and biotechnological application. Electron. J. Biotechnol. 31, 48–56. doi: 10.1016/j.ejbt.2017.11.001

[ref91] Puzenat V. Escartín J. Martelat J.-E. Barreyre T. Le Moine Bauer S. Nomikou P.. (2021). Shallow-water hydrothermalism at Milos (Greece): nature, distribution, heat fluxes and impact on ecosystems. Mar. Geol. 438:106521. doi: 10.1016/j.margeo.2021.106521

[ref92] Reeder J. Knight R. (2010). Rapidly denoising pyrosequencing amplicon reads by exploiting rank-abundance distributions. Nat. Methods 7, 668–669. doi: 10.1038/nmeth0910-668b, PMID: 20805793 PMC2945879

[ref93] Reyes C. Schneider D. Lipka M. Thürmer A. Böttcher M. E. Friedrich M. W. (2017). Nitrogen metabolism genes from temperate marine sediments. Mar. Biotechnol. 19, 175–190. doi: 10.1007/s10126-017-9741-0, PMID: 28283802 PMC5405112

[ref94] Roux S. Faubladier M. Mahul A. Paulhe N. Bernard A. Debroas D.. (2011). Metavir: a web server dedicated to virome analysis. Bioinformatics 27, 3074–3075. doi: 10.1093/bioinformatics/btr519, PMID: 21911332

[ref95] Schine C. M. S. Alderkamp A.-C. van Dijken G. Gerringa L. J. A. Sergi S. Laan P.. (2021). Massive Southern Ocean phytoplankton bloom fed by iron of possible hydrothermal origin. Nat. Commun. 12:1211. doi: 10.1038/s41467-021-21339-5, PMID: 33619262 PMC7900241

[ref96] Schuler C. G. Havig J. R. Hamilton T. L. (2017). Hot spring microbial community composition, morphology, and carbon fixation: implications for interpreting the ancient rock record. Front. Earth Sci. 5:97. doi: 10.3389/feart.2017.00097

[ref97] Sciutteri V. Smedile F. Vizzini S. Mazzola A. Vetriani C. (2022). Microbial biofilms along a geochemical gradient at the shallow-water hydrothermal system of Vulcano Island, Mediterranean Sea. Front. Microbiol. 13:840205. doi: 10.3389/fmicb.2022.840205, PMID: 35283854 PMC8905295

[ref98] Sievert S. M. Brinkhoff T. Muyzer G. Ziebis W. Kuever J. (1999). Spatial heterogeneity of bacterial populations along an environmental gradient at a shallow submarine hydrothermal vent near Milos Island (Greece). Appl. Environ. Microbiol. 65, 3834–3842. doi: 10.1128/AEM.65.9.3834-3842.1999, PMID: 10473383 PMC99708

[ref99] Sievert S. M. Kuever J. Muyzer G. (2000). Identification of 16S ribosomal DNA-defined bacterial populations at a shallow submarine hydrothermal vent near Milos Island (Greece). Appl. Environ. Microbiol. 66, 3102–3109. doi: 10.1128/AEM.66.7.3102-3109.2000, PMID: 10877814 PMC92119

[ref100] Sievert S. M. Vetriani C. (2012). Chemoautotrophy at deep-sea vents: past, present, and future. Oceanography 25, 218–233. doi: 10.5670/oceanog.2012.21

[ref101] Sievert S. M. Wieringa E. B. A. Wirsen C. O. Taylor C. D. (2007). Growth and mechanism of filamentous-sulfur formation by *Candidatus Arcobacter sulfidicus* in opposing oxygen-sulfide gradients. Environ. Microbiol. 9, 271–276. doi: 10.1111/j.1462-2920.2006.01156.x, PMID: 17227432

[ref102] Smirnov A. Y. Mourokh L. G. Nori F. (2009). Kinetics of proton pumping in cytochrome c oxidase. J. Chem. Phys. 130:235105. doi: 10.1063/1.3155213, PMID: 19548766

[ref103] Springael D. Top E. M. (2004). Horizontal gene transfer and microbial adaptation to xenobiotics: new types of mobile genetic elements and lessons from ecological studies. Trends Microbiol. 12, 53–58. doi: 10.1016/j.tim.2003.12.010, PMID: 15040322

[ref104] Tang K. Liu K. Jiao N. Zhang Y. Chen C.-T. A. (2013). Functional metagenomic investigations of microbial communities in a shallow-sea hydrothermal system. PLoS One 8:e72958. doi: 10.1371/journal.pone.0072958, PMID: 23940820 PMC3735525

[ref105] Tarasov V. G. (2006). “Effects of shallow-water hydrothermal venting on biological communities of coastal marine ecosystems of the Western Pacific” in Advances in Marine Biology. eds. Southward A. J. Young C. M. Fuiman L. A. (London, U.K.: Academic Press (Elsevier)) vol. 50, 267–421. doi: 10.1016/S0065-2881(05)50004-X 16782453

[ref106] Tarasov V. G. Gebruk A. V. Mironov A. N. Moskalev L. I. (2005). Deep-sea and shallow-water hydrothermal vent communities: two different phenomena? Chem. Geol. 224, 5–39. doi: 10.1016/j.chemgeo.2005.07.021

[ref107] Truong D. T. Franzosa E. A. Tickle T. L. Scholz M. Weingart G. Pasolli E.. (2015). MetaPhlAn2 for enhanced metagenomic taxonomic profiling. Nat. Methods 12, 902–903. doi: 10.1038/nmeth.3589, PMID: 26418763

[ref108] Vetriani C. Voordeckers J. W. Crespo-Medina M. O’Brien C. E. Giovannelli D. Lutz R. A. (2014). Deep-sea hydrothermal vent *Epsilonproteobacteria* encode a conserved and widespread nitrate reduction pathway (nap). ISME J. 8, 1510–1521. doi: 10.1038/ismej.2013.246, PMID: 24430487 PMC4069388

[ref109] Vigil-Stenman T. Ininbergs K. Bergman B. Ekman M. (2017). High abundance and expression of transposases in bacteria from the Baltic Sea. ISME J. 11, 2611–2623. doi: 10.1038/ismej.2017.114, PMID: 28731472 PMC5649170

[ref110] Wang L. Cheung M. K. Kwan H. S. Hwang J.-S. Wong C. K. (2015). Microbial diversity in shallow-water hydrothermal sediments of Kueishan Island, Taiwan as revealed by pyrosequencing. J. Basic Microbiol. 55, 1308–1318. doi: 10.1002/jobm.201400811, PMID: 26132902

[ref111] Wang L. Cheung M. K. Liu R. Wong C. K. Kwan H. S. Hwang J.-S. (2017). Diversity of total bacterial communities and chemoautotrophic populations in sulfur-rich sediments of shallow-water hydrothermal vents off Kueishan Island, Taiwan. Microb. Ecol. 73, 571–582. doi: 10.1007/s00248-016-0898-2, PMID: 27909749

[ref112] Wang Q. Garrity G. M. Tiedje J. M. Cole J. R. (2007). Naive Bayesian classifier for rapid assignment of rRNA sequences into the new bacterial taxonomy. Appl. Environ. Microbiol. 73, 5261–5267. doi: 10.1128/AEM.00062-07, PMID: 17586664 PMC1950982

[ref113] Wang S. Jiang L. Hu Q. Cui L. Zhu B. Fu X.. (2021). Characterization of *Sulfurimonas hydrogeniphila* sp. nov., a novel bacterium predominant in deep-sea hydrothermal vents and comparative genomic analyses of the genus Sulfurimonas. Front. Microbiol. 12:626705. doi: 10.3389/fmicb.2021.626705, PMID: 33717015 PMC7952632

[ref114] Weissgerber T. Dobler N. Polen T. Latus J. Stockdreher Y. Dahl C. (2013). Genome-wide transcriptional profiling of the purple sulfur bacterium *Allochromatium vinosum* DSM 180T during growth on different reduced sulfur compounds. J. Bacteriol. 195, 4231–4245. doi: 10.1128/jb.00154-13, PMID: 23873913 PMC3754752

[ref115] Wenzhöfer F. Holby O. Glud R. N. Nielsen H. K. Gundersen J. K. (2000). In situ microsensor studies of a shallow water hydrothermal vent at Milos, Greece. Mar. Chem. 69, 43–54. doi: 10.1016/S0304-4203(99)00091-2

[ref116] Westcott S. L. Schloss P. D. (2015). De novo clustering methods outperform reference-based methods for assigning 16S rRNA gene sequences to operational taxonomic units. PeerJ 3:e1487. doi: 10.7717/peerj.1487, PMID: 26664811 PMC4675110

[ref117] Wirsen C. O. Sievert S. M. Cavanaugh C. M. Molyneaux S. J. Ahmad A. Taylor L. T.. (2002). Characterization of an autotrophic sulfide-oxidizing marine *Arcobacter* sp. that produces filamentous sulfur. Appl. Environ. Microbiol. 68, 316–325. doi: 10.1128/AEM.68.1.316-325.2002, PMID: 11772641 PMC126556

[ref118] Wu S. Zhu Z. Fu L. Niu B. Li W. (2011). WebMGA: a customizable web server for fast metagenomic sequence analysis. BMC Genomics 12:444. doi: 10.1186/1471-2164-12-444, PMID: 21899761 PMC3180703

[ref119] Xie S. Wang S. Li D. Shao Z. Lai Q. Wang Y.. (2021). *Sulfurovum indicum* sp. nov., a novel hydrogen- and sulfur-oxidizing chemolithoautotroph isolated from a deep-sea hydrothermal plume in the northwestern Indian Ocean. Int. J. Syst. Evol. Microbiol. 71:004748. doi: 10.1099/ijsem.0.004748 33734956

[ref120] Yücel M. (2013). Down the thermodynamic ladder: a comparative study of marine redox gradients across diverse sedimentary environments. Estuar. Coast. Shelf Sci. 131, 83–92. doi: 10.1016/j.ecss.2013.07.013

[ref121] Yücel M. Gartman A. Chan C. S. Luther G. W. (2011). Hydrothermal vents as a kinetically stable source of iron-sulphide-bearing nanoparticles to the ocean. Nat. Geosci. 4, 367–371. doi: 10.1038/ngeo1148

[ref122] Yücel M. Sievert S. M. Vetriani C. Foustoukos D. I. Giovannelli D. Le Bris N. (2013). Eco-geochemical dynamics of a shallow-water hydrothermal vent system at Milos Island, Aegean Sea (eastern Mediterranean). Chem. Geol. 356, 11–20. doi: 10.1016/j.chemgeo.2013.07.020

