# Supplementary figures and images for: Hydrodynamic flow and benthic boundary layer interactions shape the microbial community in Milos shallow water hydrothermal vents
Source: Front Microbiol. 2025 Aug 29;16:1649514. doi: 10.3389/fmicb.2025.1649514 (PMC12425947; doi:10.3389/fmicb.2025.1649514)

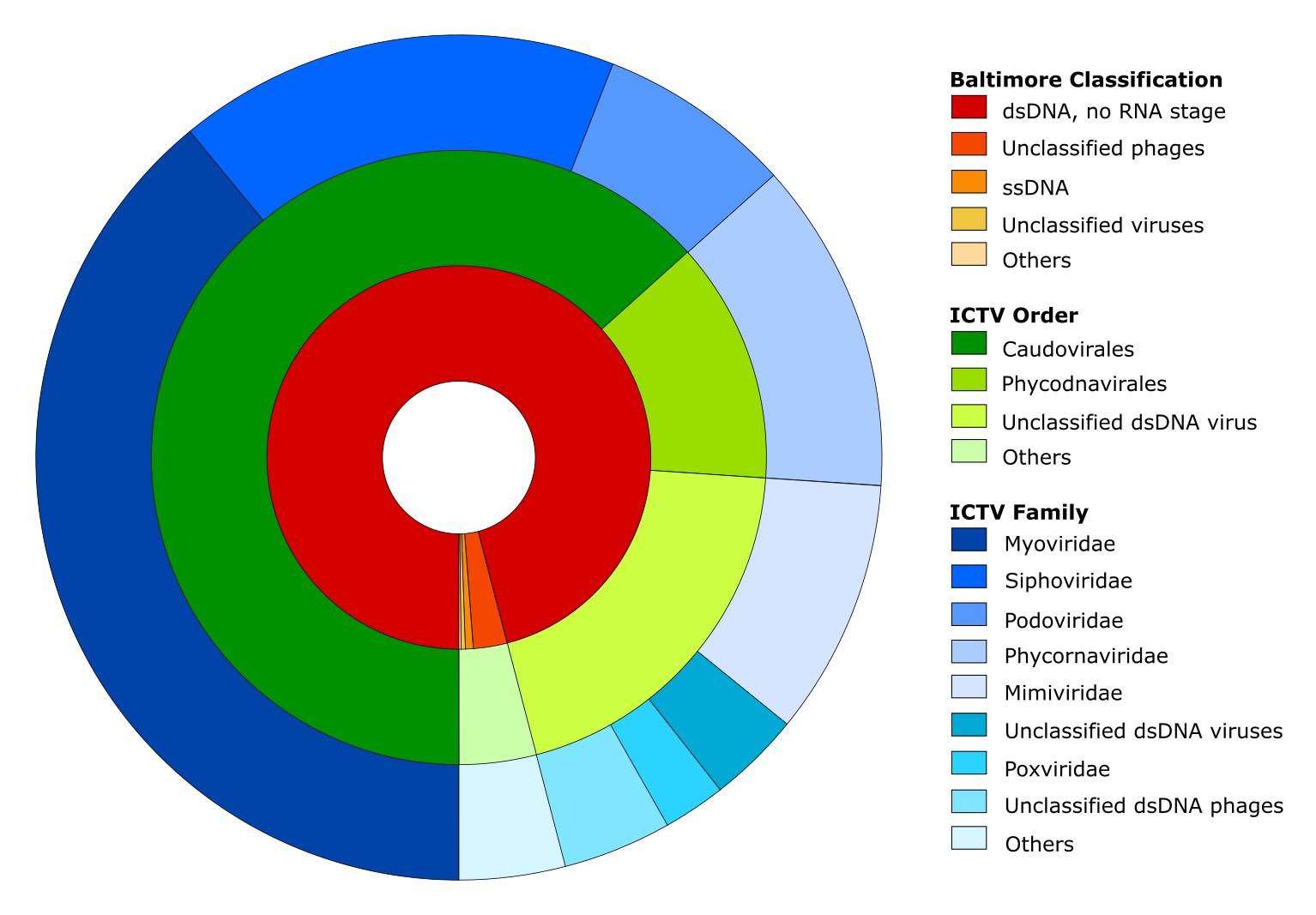

Supplement: SUPPLEMENTARY FIGURE S2 — Viral diversity in the microbial mat metagenome classified according to the Baltimore classification (inner circle), and the International Commission on Viral Taxonomy classification at the Order (median circle) and Family (outer circle) level. A total of 30,891 sequences were classified using MetaVIR with an e-value cutoff of 10−5 (a total of 50,050 sequences were classified using a less conservative e-value cutoff of 10−3). [file Image_2.jpeg]

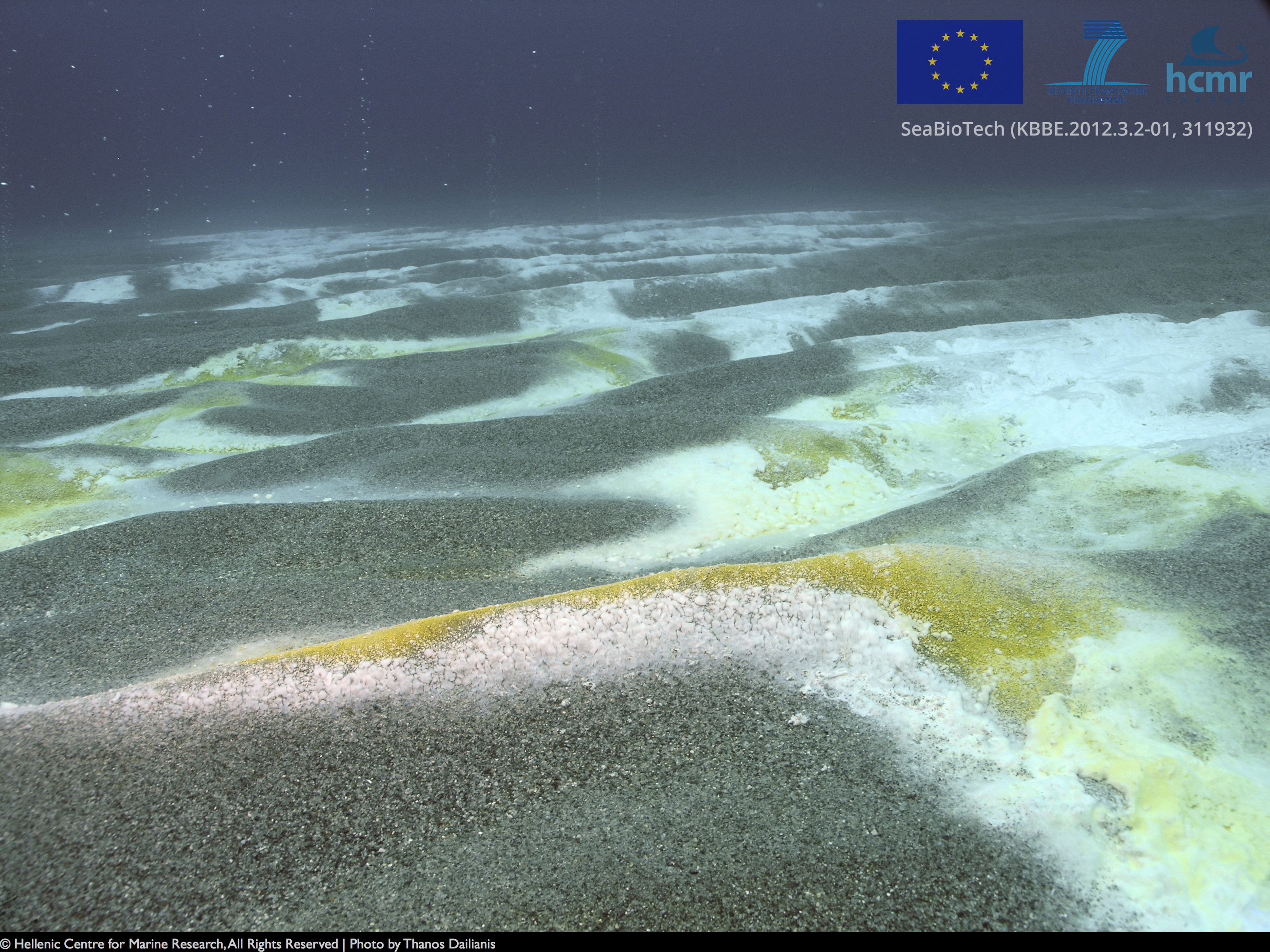

Supplement: SUPPLEMENTARY FIGURE S3 — Photograph of microbial mats at the hydrothermal venting area off Milos Island, Greece. The image shows a series of sand ripples coated with microbial mats displaying distinct surface coloration gradients: white, yellow, light reddish/orangish and brownish, with yellow mats often overlying white mats along ripple flanks. These mat colors reflect differences in microbial community composition and are consistent with stratified layering represented in Figure 7B. The coloration and distribution are influenced by venting intensity and hydrodynamic flow conditions. Image credit: Thanos Dailianis/Hellenic Centre for Marine Research (SeaBioTech project, FP7-KBBE.2012.3.2-01). [file Image_3.jpeg]
